# Mapping major SARS-CoV-2 drug targets and assessment of druggability using computational fragment screening: Identification of an allosteric small-molecule binding site on the Nsp13 helicase

**DOI:** 10.1371/journal.pone.0246181

**Published:** 2021-02-17

**Authors:** Matthew R. Freidel, Roger S. Armen

**Affiliations:** Department of Pharmaceutical Sciences, College of Pharmacy, Thomas Jefferson University, Philadelphia, Pennsylvania, United States of America; University of Calgary, CANADA

## Abstract

The 2019 emergence of, SARS-CoV-2 has tragically taken an immense toll on human life and far reaching impacts on society. There is a need to identify effective antivirals with diverse mechanisms of action in order to accelerate preclinical development. This study focused on five of the most established drug target proteins for direct acting small molecule antivirals: Nsp5 Main Protease, Nsp12 RNA-dependent RNA polymerase, Nsp13 Helicase, Nsp16 2’-O methyltransferase and the S2 subunit of the Spike protein. A workflow of solvent mapping and free energy calculations was used to identify and characterize favorable small-molecule binding sites for an aromatic pharmacophore (benzene). After identifying the most favorable sites, calculated ligand efficiencies were compared utilizing computational fragment screening. The most favorable sites overall were located on Nsp12 and Nsp16, whereas the most favorable sites for Nsp13 and S2 Spike had comparatively lower ligand efficiencies relative to Nsp12 and Nsp16. Utilizing fragment screening on numerous possible sites on Nsp13 helicase, we identified a favorable allosteric site on the N-terminal zinc binding domain (ZBD) that may be amenable to virtual or biophysical fragment screening efforts. Recent structural studies of the Nsp12:Nsp13 replication-transcription complex experimentally corroborates ligand binding at this site, which is revealed to be a functional Nsp8:Nsp13 protein-protein interaction site in the complex. Detailed structural analysis of Nsp13 ZBD conformations show the role of induced-fit flexibility in this ligand binding site and identify which conformational states are associated with efficient ligand binding. We hope that this map of over 200 possible small-molecule binding sites for these drug targets may be of use for ongoing discovery, design, and drug repurposing efforts. This information may be used to prioritize screening efforts or aid in the process of deciphering how a screening hit may bind to a specific target protein.

## Introduction

The societal and economic impacts of the SARS-CoV-2 virus are undeniable [1–4]. In the earliest months of the pandemic, some of the most important information to inform pharmacotherapy and immediate drug repurposing efforts was from previous research into the 2002 SARS-CoV, and the 2012 MERS coronavirus epidemics, as these viruses share many characteristics with the current SARS-CoV-2 virus [5, 6]. Unfortunately, no small-molecule drug therapy to treat Coronaviruses emerged from these earlier epidemics [7], though several small-molecule drugs and other biological (antibody) therapeutic agents [8] are currently being investigated for potential efficacy against SARS-CoV-2 [9–11]. With the attention SARS-CoV-2 has garnered, a variety of innovative approaches have been considered, even including the successful computational design of picomolar miniprotein inhibitors of the Spike ectodomain trimer [12]. In our study, we cast some focus on a potential target that has not received as much attention, Non-Structural Protein 13 (Nsp13). The SARS-CoV-2 helicase is a particularly attractive target, as it is well conserved amongst other viruses in the nidovirus family and a successful antiviral molecule may be therapeutically useful against a future Coronavirus, or other members of the nidovirales family [13].

SARS-CoV-2 is related to the original SARS virus that emerged in 2003 [5, 6]. Both come from the β-Coronavirus B-lineage and are similar enough in sequence that early homology modeling efforts were reliable enough to model the majority of the most important viral protein drug targets with reasonable confidence [14, 15]. During the time frame of February to April 2020, new structures of almost all of the SARS-CoV-2 major drug target proteins had been solved through the efforts of numerous groups worldwide and the Center for Structural Genomics of Infectious Diseases (CSGID)) [16]. Here, we take advantage of these rapid advances in our structural knowledge of these drug targets [17, 18] and explore a large number of possible small-molecule binding sites that could be targeted by virtual screening. In this comparative study, we map, evaluate, and prioritize 200 possible small-molecule binding sites on important SARS-CoV-2 target proteins.

### Pharmacophore and solvent mapping strategies

In drug discovery, one of the most useful and transformative concepts is that of the pharmacophore [19–21]. Although many polar functional groups (e.g. hydroxyls, basic amines, carboxylic acids, etc.) are important pharmacophore groups for forming specific protein-ligand interactions and determining ligand binding selectivity, an aromatic (benzene) pharmacophore is arguably the most common in drug structures and one of the greatest relative contributions to the free energy of binding (ΔG_bind_) [22–24]. In our study, it is important to consider that a benzene pharmacophore may also be a weak replacement for a more favorable non-planar lipophilic group, such a cyclohexyl, cyclopentyl, or even possibly an isopropyl group. Numerous “solvent mapping” computational approaches have been pioneered and optimized in the past to identify the location where a specific functional group may bind to a protein with specificity. The original Multi-Copy Simultaneous Search (MCSS) strategy was an elegant way for mapping a specific site in detail for a minimum subset of highly informative pharmacophores [25–27]. Numerous extensions of and refinement of such approaches, such as Site Identification by Ligand Competitive Saturation (SILCS) [28, 29] or WATERMAP [30–33] have been developed and are extremely useful for computational structure-based-drug-design (SBDD). Simultaneously, as the field of Protein-Protein-Interactions (PPIs) emerged [34–36], numerous computational methods were developed to detect the presence of hydrophobic patches or thermodynamically favorable binding “Hot Spots” on the surface of a given protein [37–40]. In our present work, we use a simple workflow of solvent mapping and free energy calculations to identify and prioritize which binding sites are most favorable for an aromatic pharmacophore. These sites are then additionally characterized using computational fragment screening with a fragment library specifically designed to elaborate and optimize an aromatic (benzene) pharmacophore.

### Ligand efficiency and druggability

Two decades of advances in experimental fragment based screening efforts have redefined how we approach evaluating the “druggability” of specific protein targets and small-molecule binding sites [41–43]. These approaches are based on analysis of binding sites or the physiochemical properties of compounds that bind to them (screening hits, fragment ligands, lead compounds, and clinical candidates) with available activity data or biophysical binding data [41]. The concept of ligand efficiency (ΔG_bind_ / HA), or ΔG_bind_ divided by the number of heavy atoms (HA), has been widely accepted and utilized by those who perform fragment screening as a metric of comparing fragment hits and screening data [44–48]. Fragment hits with high ligand efficiency (LE) and other favorable compound properties may become attractive starting points for optimization, depending on existing chemical matter and/or details of synthetic feasibility. Reported experimental fragment screening data comparing protein targets have shown significant differences in fragment hit rates and LE between different target proteins [41, 49–50]. These observations likely have their physical basis in differences in binding site architecture and the physiochemical properties of the residues forming these sites [42, 43]. For example, several studies have reported that some PPI targets have much lower hit rates and LE compared to other major structural classes of drug targets (proteases, kinases, nuclear hormone receptors, etc.,) [49, 50]. A retrospective review of numerous lead optimization campaigns to clinical candidates recently highlighted the importance of ligand efficiency (LE) and lipophilic ligand efficiency (LLE) in the trajectory of medicinal chemistry optimizations from lead compound to clinical candidates [48]. Prior to any investment in a specific experimental screening or medicinal chemistry optimization strategy, a preliminary computational assessment may be performed to assess that strategy. Calculated LE values may be used to make important decisions in drug discovery: What protein is optimal to target? What binding site is optimal to target? Of several possible lead compound optimizations strategies, which is most likely to lead to chemical series of derivatives with the highest LE, LLE or other optimal physiochemical properties?

### Targeting the Nsp 13 helicase

**Fig 1:**
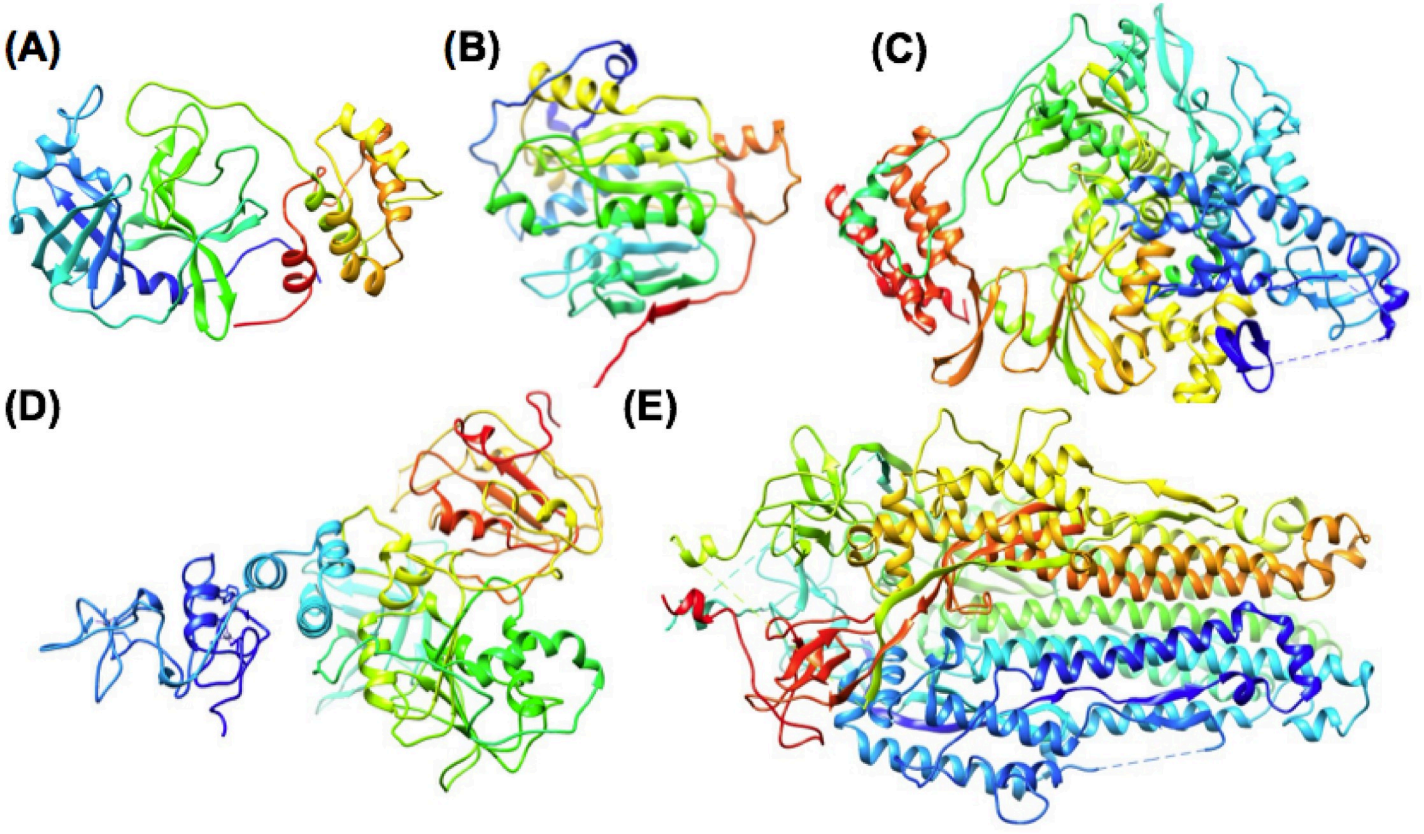
Major SARS-CoV-2 drug target proteins analyzed using pharmacophore mapping and computational fragment screening. (A) Nsp5 Mprot (B) Nsp16 2’-O MT, (C) Nsp12 RdRp (D) Nsp13 Helicase (E) S2 Spike. Protein structures are shown using ribbon diagram with RGB (red-green-blue) rainbow coloring from the N-terminus (blue) to the C-terminus (red).

The Nsp12, RNA dependent RNA Polymerase (RdRp) has been one of the primary focuses for potential SARS-CoV2 drug development. The RNA polymerase has been an established drug target for decades as extensive research has focused on developing improved inhibitors for HIV, HCV, influenza and other viruses [51–54]. Even in our investigation, we have found it to be an excellent target due to numerous thermodynamically favorable binding sites. The five SARS CoV-2 drug target proteins that are the focus of this study are shown in (Fig 1). The Nsp13 helicase was of interest to us due it being one of the least-studied targets for coronavirus and we thought it held undervalued therapeutic potential. In HCV drug development, extensive fragment screening campaign data with complementary structural studies demonstrated that the HCV NS3 protease / helicase bifunctional enzyme was capable of binding fragment ligands at 4 distinct small-molecule binding sites [55, 56]. From similar fragment screening data and structural studies for numerous structural classes of protein targets, the authors demonstrated that some protein targets may exhibit up to 3 to 4 distinct fragment ligand binding sites [56]. Thus, using the recently solved structure of the SARS-CoV helicase [57], our initial studies were motivated to identify thermodynamically favorable small-molecule binding sites other than the ATPase active site.

The viral helicase is largely conserved amongst the order nidovirales, indicating that targeting the helicase may offer a broader range of therapeutic uses than other viral proteins which have greater sequence variation [13, 58, 59]. β-coronavirus helicase enzymes also exhibit greater sequence similarity between closely related viruses (SARS-CoV, MERs, SARS-CoV-2, etc.,) compared to other viral target proteins in our study. In the sequence of SARS-CoV-2, compared to SARS-CoV, the Nsp13 helicase (ID = 99.8%) exhibits the greatest sequence similarity, compared to Nsp5 main protease (Mpro) (ID = 96.1%), Nsp12 RdRp (ID = 96.4%), or Nsp16 (ID = 93.3%). Greater divergence in sequence is observed for the Spike protein (ID = 76.8%), but our study focuses on the shorter S2 segment (residues: 711–1147) with a higher level of sequence conservation (ID = 88.2%). Using a pharmacophore mapping and fragment screening approach we are able to narrow down the areas of interest and find the binding sites that are most favorable and may offer the greatest therapeutic potential.

## Materials and methods

### Experimental structures of SARS-CoV-2 proteins

This section describes which experimental protein structures were used for computational chemistry analysis and how these proteins were prepared for solvent mapping or molecular docking calculations using CHARMM [60]. All initial work on Nsp13 helicase utilized the X-ray structure (6jyt.pdb) of the SARS-CoV virus which is only one residue different (>99% identity) to the SARS-CoV-2 virus [57]. After pharmacophore mapping and fragment screening using the full-length (res:1–596) structure of the A subunit (6jytA), new structures of the SARS-CoV-2 helicase became available and were used in subsequent analysis and fragment screening. These included the cryoEM structure of the helicase in the replicase complex (6xez.pdb) [61] and then the high-resolution 2.8 Å crystal structure of the helicase dimer (6zsl.pdb) [62]. In analysis of Nsp13 N-terminal Zinc-Binding-Domain (ZBD) binding sites, initially identified from (6jytA.pdb), subsequent cross-docking analysis was performed by superimposing the coordinates of new structures onto the reference structure (01_6jytA), thus creating 5 new reference conformations for cross-docking i.e. 02_6jytB, 03_6xezE, 04_6xezF, 05_6zslA, 06_6zslB. In this way, six independent structural snapshots of the domain in different conformational states were assessed using the pharmacophore mapping and fragment screening workflow.

Studies of the Nsp 5 main protease (Mpro) used a structure of a non-covalent broad spectrum inhibitor (6w63.pdb) [63]. This structure was selected as a representative structure of a moderately sized ligand that utilized P1, P2 and P3 substrate recognition sites [63]. In pharmacophore mapping and docking, the full-length (res:1–305) structure of the A subunit (6w63.pdb) were used for all calculations removing water and all ligand atoms from the inhibitor (X77). In subsequent analysis, after pharmacophore mapping and docking, several additional structures were accessed for analysis (6ynq.pdb) (5rgz.pdb) (5rf3.pdb) (5r81.pdb) [64–67]. A search of available structures of the protease identified three crystal structures (5ree.pdb) (5reg.pdb) (5rfc.pdb) that corroborated “minor” binding sites that were outside of the protease active site [68–70].

Studies of Nsp 4, Papin-like protease did not use the full biological assembly, but only the full-length (res:1–320) D subunit monomer of Nsp4 in complex with the peptide inhibitor VIR215 (6wx4.pdb) [71]. Similar to other proteins, bound water atoms and ligand atoms were removed prior to solvent mapping and docking. Studies of Nsp 12 RdRp, used the CryoEM complex of Nsp12 with Nsp7 and Nsp8 and the small-molecule Remdesivir (7bv2.pdb) [72]. For analysis, the available coordinates of Nsp12 RdRp were retained, but the coordinates of the other proteins in the complex, Nsp7 and Nsp8 were removed, which also allows an assessment of these protein-protein interaction sites. Studies of Nsp15 used the X-ray structure in complex with tipiracil (6wxc.pdb) [73]. Structural studies of Nsp16, used the complex bound to sinefungin and Nsp10 (6wkq.pdb), removing the coordinates of Nsp10, which allows an assessment of these protein-protein interaction sites [74]. Studies of the Spike protein, used the 3.2 Å CryoEM structure (6vyb.pdb) of the full-length SARS-CoV-2 Spike protein where the ectodomain was in the “closed” state [75]. However, our analysis was focused only on the trimeric Spike S2 segment, modeling only the trimeric structure of residues: 711–1147. For the Spike S2 segment, the trimeric structure was modeled without any of the glycosylation’s on the surface of the protein. Additional details regarding regions of S2 Spike structure deviation, or the effect of the lack of glycosylation on the surface are discussed in the results section.

### Pharmacophore mapping, docking and free energy calculations

**Fig 2:**
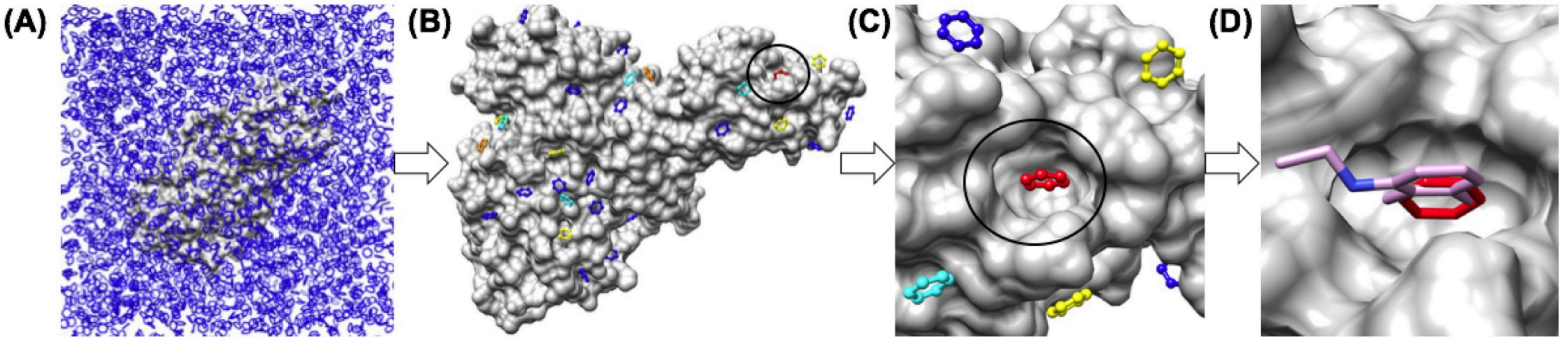
Overview of pharmacophore mapping and computational fragment screening. (A) Nsp13 helicase solvent mapping simulations with benzene solvent. (B) TOP50 sites are identified from analysis (hydrophobic contacts & geometric clustering). (C) CHARMM-based free energy calculations are utilized to calculate the free energy of the TOP50 sites and re-rank the list of aromatic pharmacophore sites by ΔG. Site 01 for the Nsp13 helicase is shown (red). (D) Fragment library screening is performed using the Site 01 aromatic pharmacophore a center-of-mass reference. Ligand efficiencies are then calculated from fragment screening data.

This section describes the workflow that utilized to identify the TOP25 or TOP50 possible aromatic pharmacophore (benzene) binding sites and how docking and free energy calculations are performed using CHARMM [58]. This workflow is summarized in (Fig 2), using the Nsp13 helicase as a representative example of the process. Starting with the edited protein structures, CHARMM-GUI [76, 77] multicomponent assembler was used to generate protein models solvated in boxes of pure benzene (with no other co-solvents) for solvent mapping molecular dynamics (MD) sampling. Most of the proteins were analyzed using a 100 Å (dimension along the X axis) cubic box. These proteins included, Nsp4 (6wx4.pdb), Nsp5 mProt (6w63.pdb), Nsp12 (7bv2.pdb), Nsp13 (6jyt.pdb), Nsp15 (6wxc.pdb) and Nsp16 (6wkq.pdb), all prepared as described previously. For proteins solvated using a 100 Å cubic box, 3283 benzene molecules were added to the box to a post-mixing concentration of 5.452 (mol/L). The elongated shape of the trimeric S2 spike protein (res:711–1147) required solvation using a slightly larger 125 Å (dimension along the X axis) cubic box, where 6413 benzene molecules were added to the box to the same post-mixing concentration of 5.452 (mol/L). For molecular dynamics (MD) simulations of these solvated complexes, the topology and parameter files associated with the CHARMM36 potential function [78] for proteins were used along with the corresponding parameters for a benzene molecule. All of these components were processed with CHARMM-GUI [76, 77] multicomponent assembler in order to set up optimal parameters for MD simulations with these systems at a temperature of 300 K. Periodic boundary conditions and Partial-mesh Ewald (PME) summation methods were utilized for electrostatic calculations with a 12 Å non-bonded cutoff. A force-based switching function is used to smooth out the non-bonded Lennard-Jones potential it approaches the cutoff distance from 10 to 12 Å [78]. A 10.0 (Kcal/mol/atom) harmonic restraining force was applied to all protein heavy atom backbone and side chain atoms during equilibration and dynamics to retain crystallographic and “native-like” structure of the protein, while surrounded by an entirely unphysical non-polar solvent. NVT dynamics were performed at a temperature of 300 K using a 1.0 fs time step to minimize integration error and reinforce the restraints on the protein structure. The system was slowly heated to temperature over 10 ps and then 500 ps of dynamics were performed to allow benzene solvent molecules to sample various conformations on the restrained protein surface.

Production MD trajectories from 100–500 ps were analyzed by calculating the number of C-C hydrophobic contacts between benzene carbons and protein atom carbons using a cutoff distance of 5.4 Å [79, 80]. In analysis of several of the datasets a threshold value of 30 contacts was determined to be a reasonable minimum number of contacts that a benzene solvent molecule would need to form with the protein surface, in order for its structure to be retained for clustering and ranking sites based on the number of hydrophobic contacts. Then, the datasets were pooled and sorted by the number of hydrophobic contacts. The representative benzene molecule with the greatest number of contacts within a cluster of representative benzene molecules (< 2.0 Å RMSD) was selected as a representative member of the cluster. Then, these sites were ranked by the number of hydrophobic contacts (Nconts) to form a list of either the TOP25 or the TOP50 aromatic pharmacophore binding sites. These benzene structures were then used as a representative X,Y,Z center of mass for docking a benzene molecule to the same site using CHARMM-based molecular docking. Following the identification of the lowest free energy (ΔG_bind_) conformation of benzene bound to the site from molecular docking, then the TOP25 or the TOP50 aromatic pharmacophore binding sites are then sorted again, ranked by (ΔG_bind_) rather than by the number of C-C hydrophobic contacts (Nconts). An important limitation of this two-step procedure is that the first selection process (based on hydrophobic contacts criteria alone) from a pure benzene solvent neglects consideration of physical desolvation penalties to binding from an aqueous solvent. However, after the sites are identified, in the second step aqueous desolvation is explicitly considered in the calculation of (ΔG_bind_). Thus, it is possible that true low energy binding sites may not be identified in the first step if a site does not satisfy the hydrophobic contact criteria.

Similar MD simulations were also performed in pure water for the Nsp13 helicase (6jyt.pdb) structure [57]. CHARMM-GUI [76, 77] multicomponent assembler was used to solvate the protein in 100 Å (dimension along the X axis) cubic box with 32318 TIP3P water molecules. For molecular dynamics (MD) simulations, the topology and parameter files associated with the CHARMM36 potential function were used [78]. Other than the use of any harmonic restraints, similar protocols for MD simulations as described above were used and CHARMM-GUI [76, 77] multicomponent assembler was used to set up optimal parameters for NVT unrestrained MD simulations of the Nsp13 helicase at 310 K in water. Simulation time, using a 1 fs timestep was extended to 500 ps for preliminary analysis. All protein structure visualization, structure modeling, 3D structural analysis, and structure-sequence based alignments were performed using USCF Chimera and ChimeraX [81, 82]. All figures were generated using USCF Chimera and ChimeraX [81, 82].

Our in-house CHARMM-based molecular docking methods were used for highly accurate predictions of small-molecule binding geometries for the final step in pharmacophore mapping and in subsequent fragment library screening. In a previously published assessment of docking accuracy, these CHARMM-based docking methods were shown to have the highest “discriminative power”to correctly predict binding geometries over diverse classes of protein-ligand interactions compared to other common scoring functions [83, 84]. Molecular docking utilized the LPDB CHARMm force field [85, 86], the Linear Interaction Energy (LIE) scoring approach to approximate the free energy of binding (ΔG_bind_) has been previously described [84, 87] and have also been specifically assessed for their use in calculating ligand efficiencies in computational medicinal chemistry applications [84]. For aromatic pharmacophore sites selected for being sufficiently favorable, computational fragment screening was performed using identified benzene coordinates as X,Y,Z center of mass reference positions. A library of fragment ligand derivatives designed to identify favorable replacements for an aromatic (benzene) pharmacophore group are docked into these reference sites. Results were characterized by free energy of binding (ΔG_bind_) and calculated ligand efficiencies where (ΔG_bind_) is divided by the number of heavy atoms within a fragment ligand.

### Aromatic heterocycle derivative fragment replacement library

**Fig 3:**
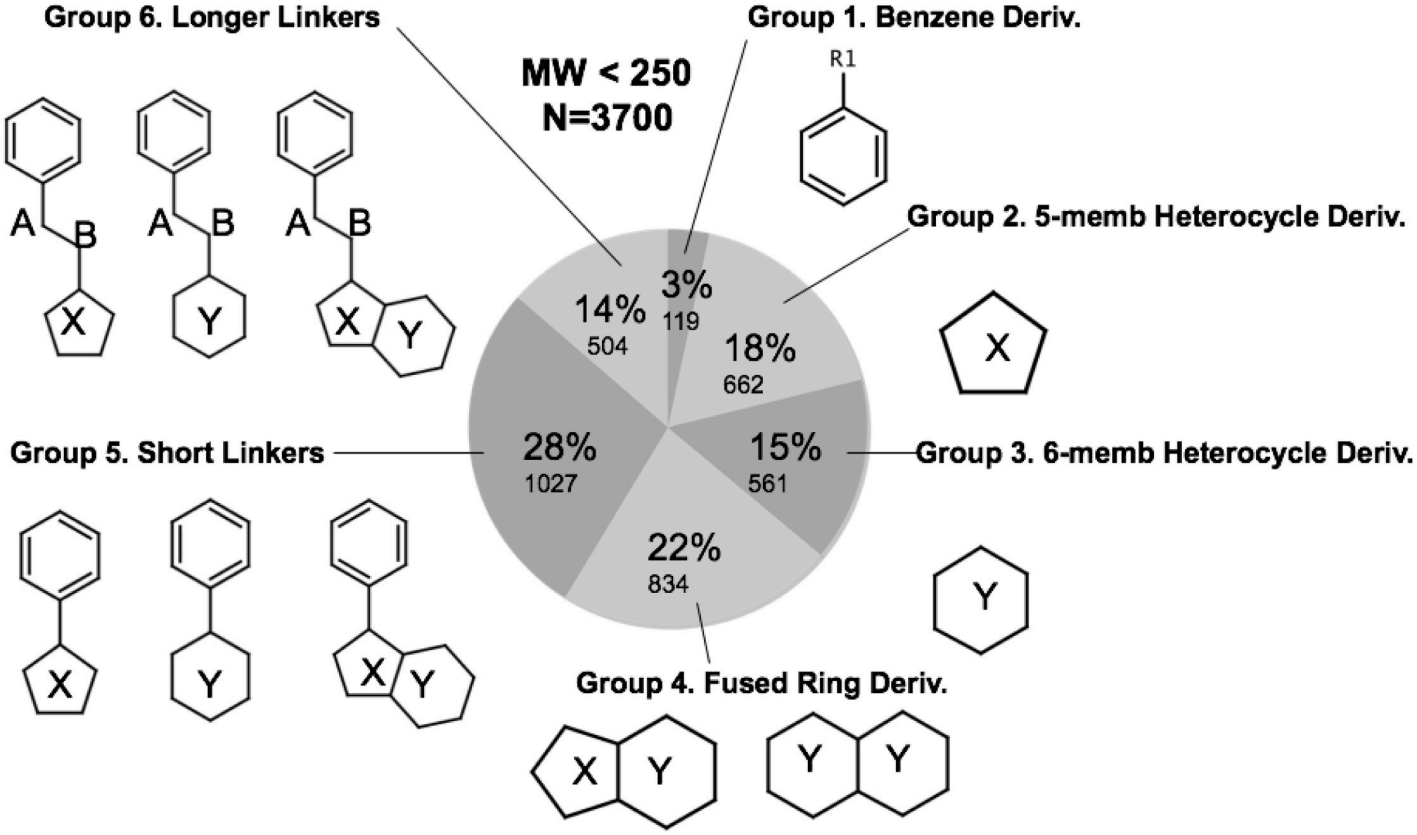
Aromatic heterocycle fragment replacement library. Additional details in supplementary information.

Our in-house library of approximately 3,700 fragment ligands was used throughout this work for fragment screening. This section describes the design, composition and physiochemical properties of this fragment library. This fragment library was first presented in 2009 for use in computational fragment screening and for elaborating and optimizing an aromatic pharmacophore group in an academic laboratory setting [88]. Every compound (cmp) in our library exists as an annotated entry in PUBCHEM [84], with the requirement that it is low in molecular weight (MW < 250). The library is designed to identify favorable fragment hits, that may be used in subsequent chemoinformatic substructure searches in PUBCHEM [89]. Although every cmp entry does have a corresponding entry in PUBCHEM, not all cmp have vendors. The composition in the library is distributed based on variations in aromatic heterocycle structure shown in (Fig 3). Some representative substructures composing groups 1 –group 6 in the library are shown in (S1 Fig in S1 File) where MW and cLogP filters were used to populate diverse and representative cmp structures for each of the “Group” designations in the library Group 1 –Group 6 (S1 Fig in S1 File) and described below. Distributions of important physiochemical properties such as molecular weight, cLogP, number of rotatable bonds are shown in (S2 Fig in S1 File).

Group 1 within the library (MW < 150) contains only substructures of benzene, and samples a diverse series of hydrophilic and hydrophobic substituted benzene derivatives. These include halogen (F, Cl, Br) and diverse alkyl hydrophobic substituents as well as several polar R-groups (phenols, aryl amines, ethers, ketones, carboxylic acids, aldehydes, esters, amides, nitro, etc.) and diverse R-group substitution patterns. The first 100 cmps of the library are denoted in this work as FRAG100 and this mini-library of benzene derivatives is also used within the current work as a small library of derivatives to compare calculated ligand efficiencies between different aromatic pharmacophore binding sites. We also refer to FRAG1000 and FRAG3700 to denote the first 1000 cmp or the entire library 3700 respectively. Group 2 (MW < 150) within the library (18%) contains only substructures of 5-membered rings and heterocycles specifically including derivatives of furans, thiophenes, pyrroles, pyrrolidines, pyrazoles, dioxolanes, oxazoles, isoxazoles, thiazoles, imidazoles, imidazolidines, etc. Group 3 within the library (MW < 150) contains only substructures of 6-membered rings and heterocycles specifically including derivatives of cyclohexyls, pyrans, dioxanes, pyridines, piperidines, morpholinos, pyrimidines, pyridazines, etc. Group 4 within the library (MW < 200) contains only substructures of various fused ring systems including derivatives of indoles, benzofurans, indazoles, benzimidizoles, purines, quinolines, quinazolines, etc. Group 5 within the library (MW < 200) contains only substructures of an aromatic pharmacophore attached by one bond to another variable R group (saturated or unsaturated aromatic group replacements). Thus, Group 5 includes derivatives of (biphenyls, 2-phenylfuran, 2-phenylpyrrole, etc). Group 6 within the library (MW < 250) requires substructures to contain a benzene connected to a specific 3-bond linkers including: alkyl (Ph-C-C-R_1_), ether (Ph-C-O-R_1_, or Ph-O-C-R_1_), amide (Ph-CONH-R_1_, or PH-NHCO-R_1_) or amine (PH-NH-C-R_1_, or Ph-C-NH-R_1_) linkers to another variable R_1_ group (saturated or unsaturated aromatic group replacements).

For this current study, to compare and characterize binding sites; a strength of this library composition is the dense sampling of cmp structure in the lower molecular weight range (100 < MW < 200) with a diverse number of cmp substructures and derivatives thereof. Due to the use of MW filters at (MW>150) and (MW<200) in some groups, the cmp selections under sampled chemical space in the specific region of (150 < MW < 175.) One other limitation of this fragment library is that sampling of compounds in the molecular weight range (200 < MW < 250) is not as thorough, as less than 14% of the library covers this range. However, in comparing optimal calculated ligand efficiencies from screening the library and using hits for follow-up substructure searches, this issue is less problematic. This is because fragment hits with more optimal efficiency are frequently on the lower end of the molecular weight range (100 < MW < 200). A more thorough sampling of small-molecule diversity on the low MW range (100 < MW < 200) is important for using fragment screening to compare ligand efficiencies. Thus, the composition and diversity of this library makes it particularly appropriate for calculation of comparative ligand efficiencies from fragment screening at aromatic pharmacophore binding sites. The low number of rotatable bonds (Nrot) in diverse representative compounds in the library (S2D Fig in S1 File) also improves overall docking accuracy of the library screening results.

## Results and discussion

### Mapping SARS-CoV-2 drug targets

The overall results from the pharmacophore mapping studies allow a comparison of predicted ΔG_bind_ for small-molecule binding sites on individual protein targets. This data also enables comparison of the most favorable binding sites on different protein targets. For the two smaller proteins Nsp5 Mpro (305 residues) and Nsp16 2’-O MT (296 residues), it was sufficient to identify and characterize the TOP25 sites. For the much larger target proteins, including the Nsp12 RdRp (930 residues), Nsp13 helicase (596 residues), and the Spike S2 segment (436 residues monomer, 1308 total residues in the trimer) it was important to extend the characterization to the TOP50 sites. The most unfavorable site in the ranked TOP25 for Nsp5 Mpro was (LE = 0.08) and for Nsp16 (LE = 0.15) respectively. In comparison, for the larger proteins the 25^th^ ranked sites (Site25) had ligand efficiencies (LE = 0.24, 0.20, 0.26) for the Nsp12 RdRp, Nsp13 helicase the S2 Spike protein, respectively. For these larger proteins, moving down the ranked list from the 25^th^ ranked site to the 50^th^ ranked site (Site50), the ligand efficiencies (LE = 0.08, 0.07, 0.16) for Nsp12, Nsp13, and the S2 Spike protein respectively converge to a similar range (LE > 0.17) for the least favorable (25^th^ ranked sites) on the smaller proteins. Therefore, we conclude that it was appropriate to analyze the TOP25 sites for the smaller proteins and the TOP50 sites for the larger proteins.

**Fig 4:**
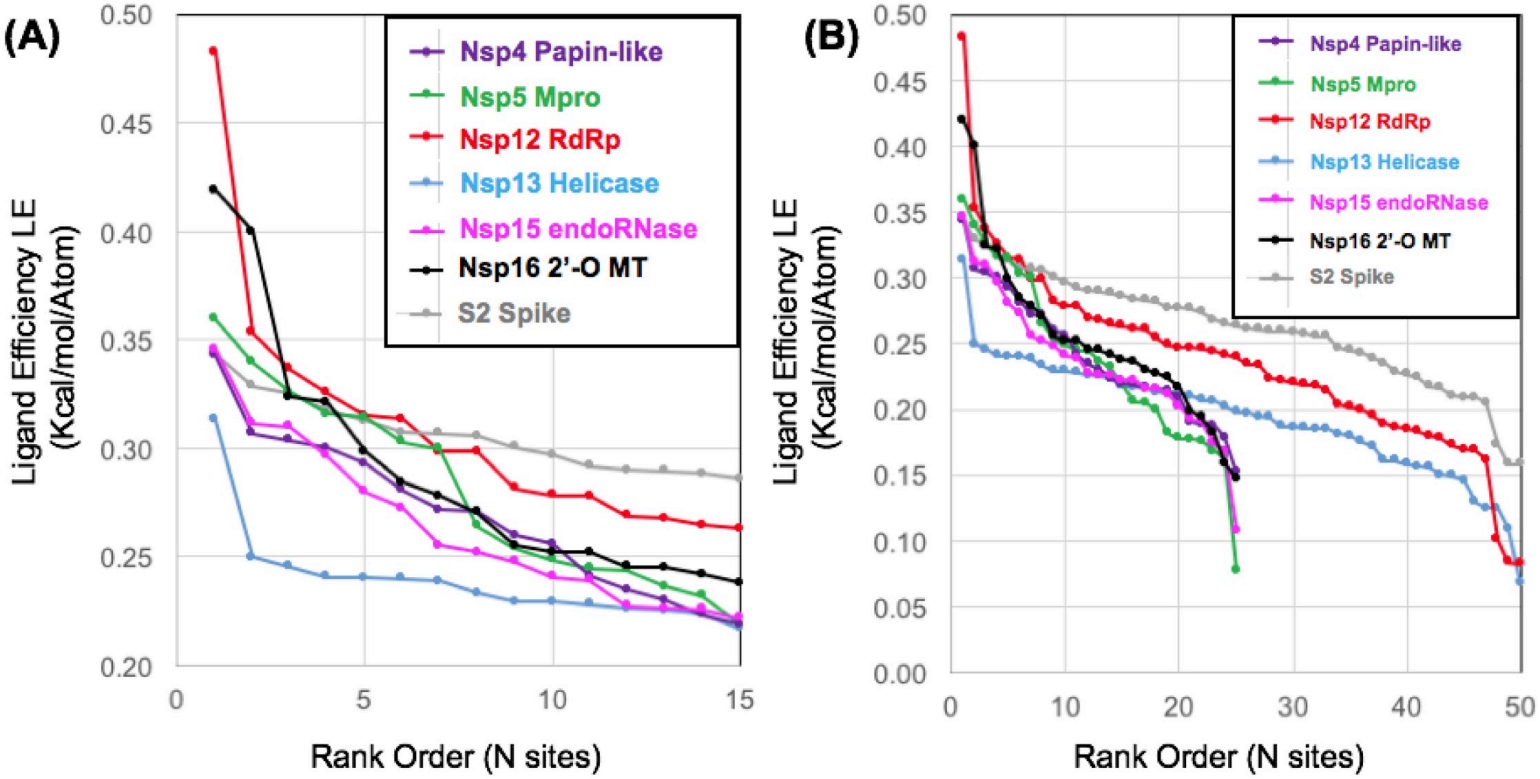
Calculated ligand efficiency comparisons of aromatic pharmacophore sites. The data is shown plotted focusing on the rank order of the first 15 most favorable sites (A) or over all of the sites characterized (B).

Overall results from the initial pharmacophore mapping step are shown in (Fig 4). The data for the structural map of aromatic pharmacophore sites and calculated ligand efficiencies are also provided as available supplementary information in the form of detailed data table (S1 Table in S1 File) and a corresponding.zip file of associated.pdb files (S2 File). Every entry in the table (S1 Table in S1 File) represents a.pdb file in the (S2 File) for aromatic pharmacophore sites on a specific target protein. From the initial pharmacophore mapping, as shown on (Fig 4), the most favorable site overall was on the Nsp12 RdRp, which contained on the order of 4–5 of some of the most favorable sites amongst the target proteins. An expected result from the pharmacophore mapping workflow was that the peptide binding site of the Nsp5 Mpro and the Nsp16 binding site for sinefungin were found to have some of the most favorable ΔG_bind_ values and calculated ligand efficiencies for all of the sites on the SARS-CoV-2 protein targets. As these protein targets are shorter in sequence and also less complex in terms of multidomain protein structure, we will describe results for the Nsp5 Mpro and Nsp16 first and then the larger proteins. The results comparing all protein targets show that Nsp5 Mpro, Nsp12 RdRp, and Nsp16 all contain two or more of the most favorable sites overall among all of the target proteins. In comparison, the Nsp13 helicase and the S2 Spike protein contained fewer favorable sites according to our initial mapping. Results comparing the most favorable sites for each protein and analysis thereof will be summarized below by protein.

**Fig 5:**
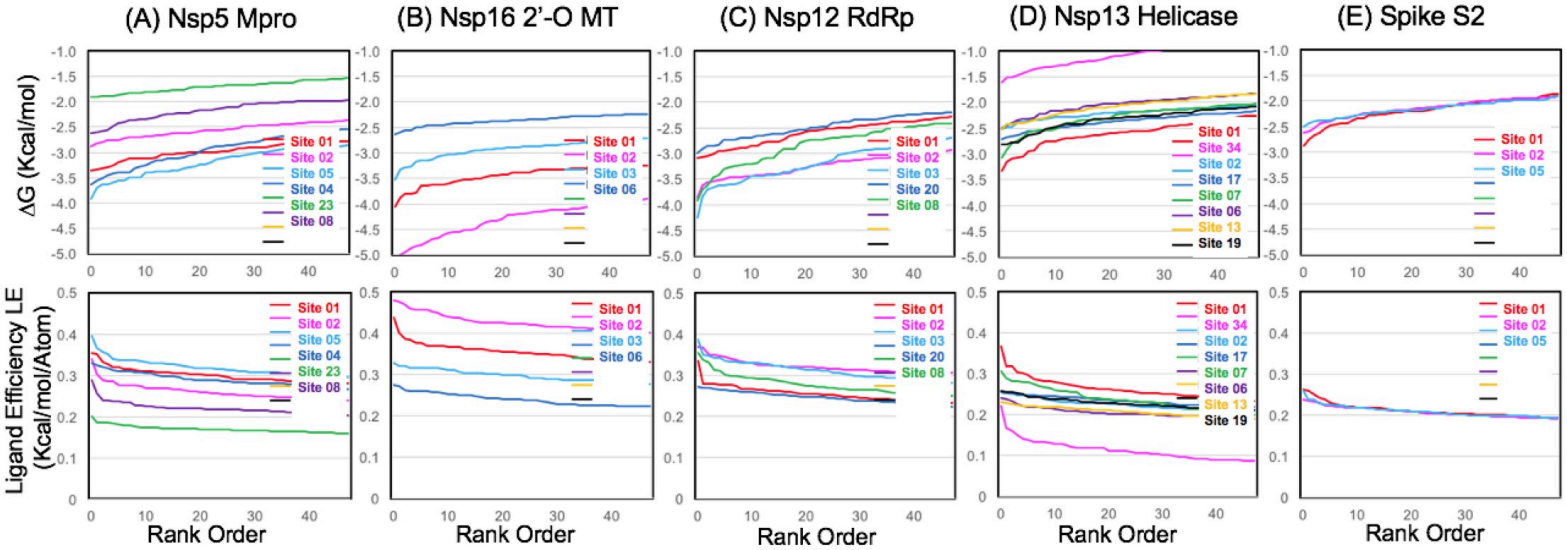
Ligand efficiency comparisons from computational fragment screening. Data is shown on the same Y-axis scale for free energy (ΔG_bind_) and ligand efficiency comparing numerous sites on different target proteins using the FRAG100 library as a benchmark: (A) Nsp5 Mpro (B) Nsp16 2’-O MT (C) Nsp12 RdRp (D) Nsp13 Helicase (E) S2 Spike.

For each target protein, once the pharmacophore mapping procedure was able to identify the most favorable sites, computational fragment screening and analysis was performed to additionally characterize and compare the most favorable sites. Calculated ligand efficiencies are shown in (Fig 5) from screening the same benchmark fragment library of aromatic pharmacophore derivatives (FRAG100) at each site. This allows a relative comparison of the so-called “druggability” of each site based on the properties of the fragment hits, which reflects structural and physiochemical properties related to the specific molecular architecture and important hydrophobic residue interacting groups that form the binding sites. Overall results from fragment screening to compare the relative ligand efficiencies of several target proteins are shown in (Fig 5). The same data are also shown in (S3 Fig in S1 File) plotting ligand efficiency as function of fragment ligand physiochemical properties. Several recent literature studies have recommended that fragment screening data should be plotted as ligand efficiency (y) plotted against N heavy atoms (x) as a new best practice [90–92]. This allows the observation (or lack thereof) of favorable ligand efficiencies over a range of increasing molecular weights. As our comparison FRAG100 library of benzene derivatives has a very narrow range of (6–11) heavy atoms and low average MW (MW < 150), it is more informative to plot our calculated ligand efficiencies (ΔG_bind_ / N heavy atoms) as a function of MW rather than HA (S3 Fig in S1 File).

### Nsp5 main protease (Mpro)

**Fig 6:**
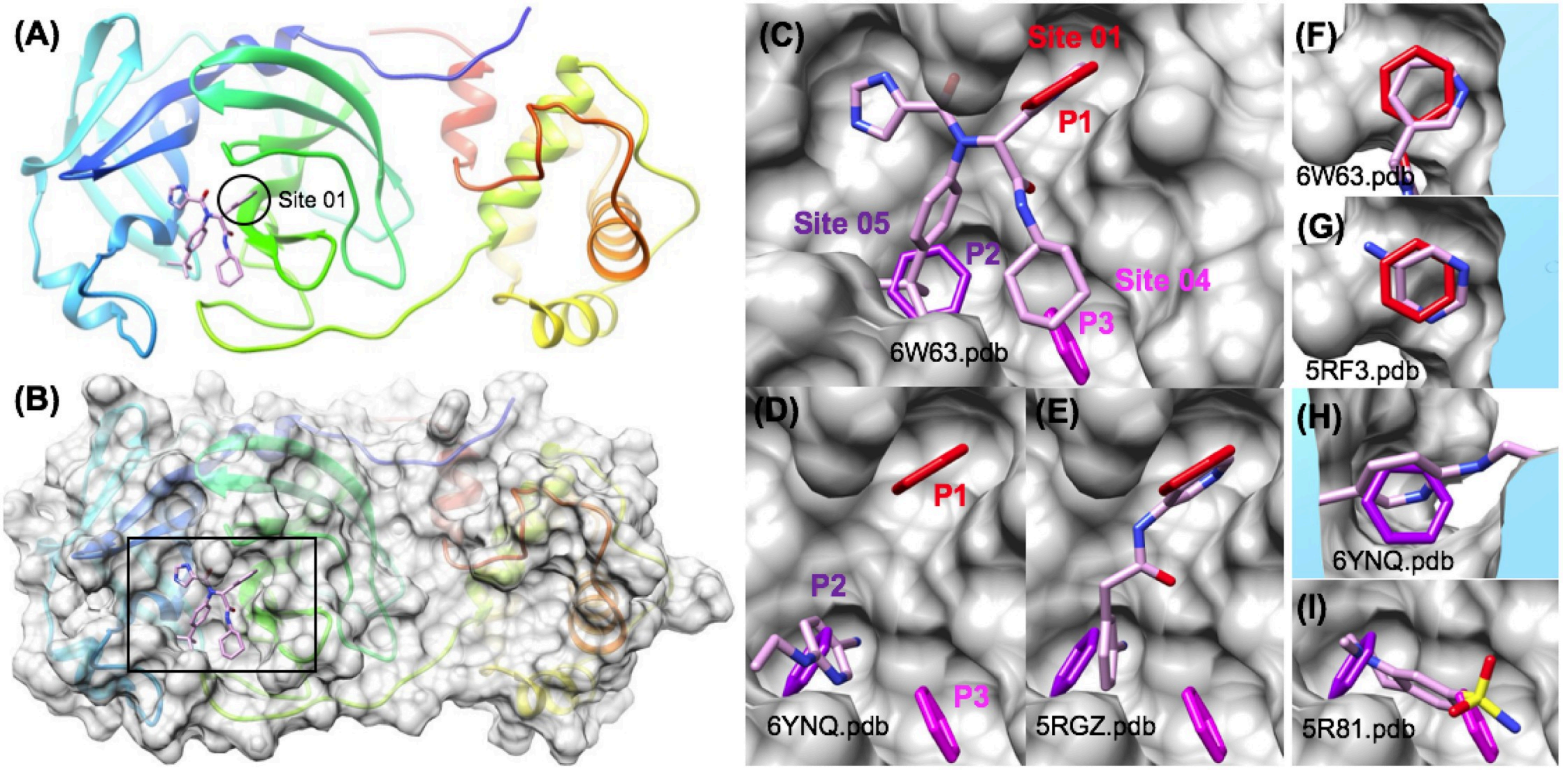
Pharmacophore mapping successfully identifies Nsp5 Mpro inhibitor substrate recognition site pharmacophores. The crystal structure of Nsp5 Mpro (6W63.pdb) bound to a broad spectrum non-covalent inhibitor is shown using ribbon diagram with rainbow coloring (A) and is shown with a transparent gray surface in (B). The three protease P1, P2, and P3 substrate recognition binding pockets were identified with favorable aromatic pharmacophores as shown for Site 01, Site 05 and Site 04 respectively (C). Several crystal structures of other fragment ligands (6YNQ.pdb, 5RGZ.pdb, 5RF3.pdb, 6YNQ.pdb, 5R81.pdb) independently confirm the position of the Site 01 (P1) and Site 05 (P2) pharmacophores shown in (D) thru (I).

Compared to the size of some of the larger protein targets, the Nsp5 Main protease (Mpro) is a relatively small protein (305 residues), and it is not surprising that the pharmacophore mapping technique was able to correctly identify what would be expected to be the three most favorable pharmacophore binding sites. The 3 most favorable peptide side chain substrate recognition sites (P1, P2, P3) were correctly mapped to aromatic (benzene) pharmacophore positions that were all ranked within the TOP5 most favorable sites (Fig 6). Numerous classes of inhibitors have been identified through virtual screening at the active site [93]. Site 01 (LE = 0.36) was the most favorable in the initial pharmacophore mapping step which is the location of the P1 substrate binding pocket as shown in (Fig 6C) and (Fig 6F) [63]. Of these sites, the next most favorable was Site 04 (LE = 0.32) which is the P3 substrate binding pocket and Site 05 (LE = 0.31) which is the P2 substrate binding pocket. The pharmacophore for Site 01 was a very good geometric match to the reference structure with low root mean squared deviation (RMSD) match of (RMSD = 0.87 Å) over six heavy atoms of the pyridyl group in the reference inhibitor as shown in (Fig 6C) and (Fig 6F) [63].

As numerous new experimental crystal structures of Nsp5 Mpro bound to fragment ligands became available, we were able to analyze the pharmacophore mapping prediction in more detail. While the geometric match of the predicted Site 01 pharmacophore to the P1 pocket substituent in the reference non-covalent broad spectrum inhibitor (6w63.pdb) [63] is very good (RMSD = 0.87 Å), there are numerous other independent crystal structures of small MW fragment ligands, such as the pyrimidin-5-amine fragment (5rf3.pdb) shown in (Fig 6G) that independently verify the exact location of this aromatic pharmacophore site at the P1 pocket [66]. As a demonstration of fragment docking accuracy (positive controls), we show that docking into the reference structure (6w63.pdb) using the Site 01 pharmacophore position is able to demonstrate correct docking retrospective predictions (RMSD < 2.0 Å) for representative P1 pocket reference fragments (S4 Fig in S1 File) [63]. Therefore, in upcoming sections focused on analysis of other target proteins such as the Nsp13 helicase, the Site 01 (P1) pocket is used as a “reference” fragment binding site for the Nsp5 Mpro. This is for two reasons, it is the best example of a lowest energy aromatic pharmacophore match (RMSD = 0.87 Å) for Nsp5 Npro and the retrospective prediction fragment docking accuracy was very good: (~80%) where predicted geometries were sufficiently close (RMSD < 2.0 Å) to correct reference fragment ligand geometries (S4 Fig in S1 File).

As shown in (Fig 6C) on the Nsp 5 Mpro, the Site 04 (P3) and Site 05 (P2) pharmacophore matches are to important functional groups for binding, but are not true “isosteric” matches to an aromatic group, but rather overlap with non-aromatic aliphatic group. The Site 05 (P2) pharmacophore superimposes with a favorable t-butyl in the reference inhibitor structure (6w63.pdb). Similarly, the Site 04 (P3) pharmacophore superimposes with part of a cyclohexyl group of the reference inhibitor (6w63.pdb). However, even though these aromatic pharmacophores (P2) and (P3) are partial matches to these aliphatic substituents of the reference inhibitor; several crystal structures of other fragment ligands (6ynq.pdb, 5rgz.pdb) independently confirm (with low RMSD) the position of the Site 05 (P2) pharmacophore as shown in (Fig 6D) and (Fig 6H). Despite the fact that few fragment ligands were found to bind in the Site 04 (P3) pocket, one example (5r81.pdb) shown in (Fig 6I).

**Fig 7:**
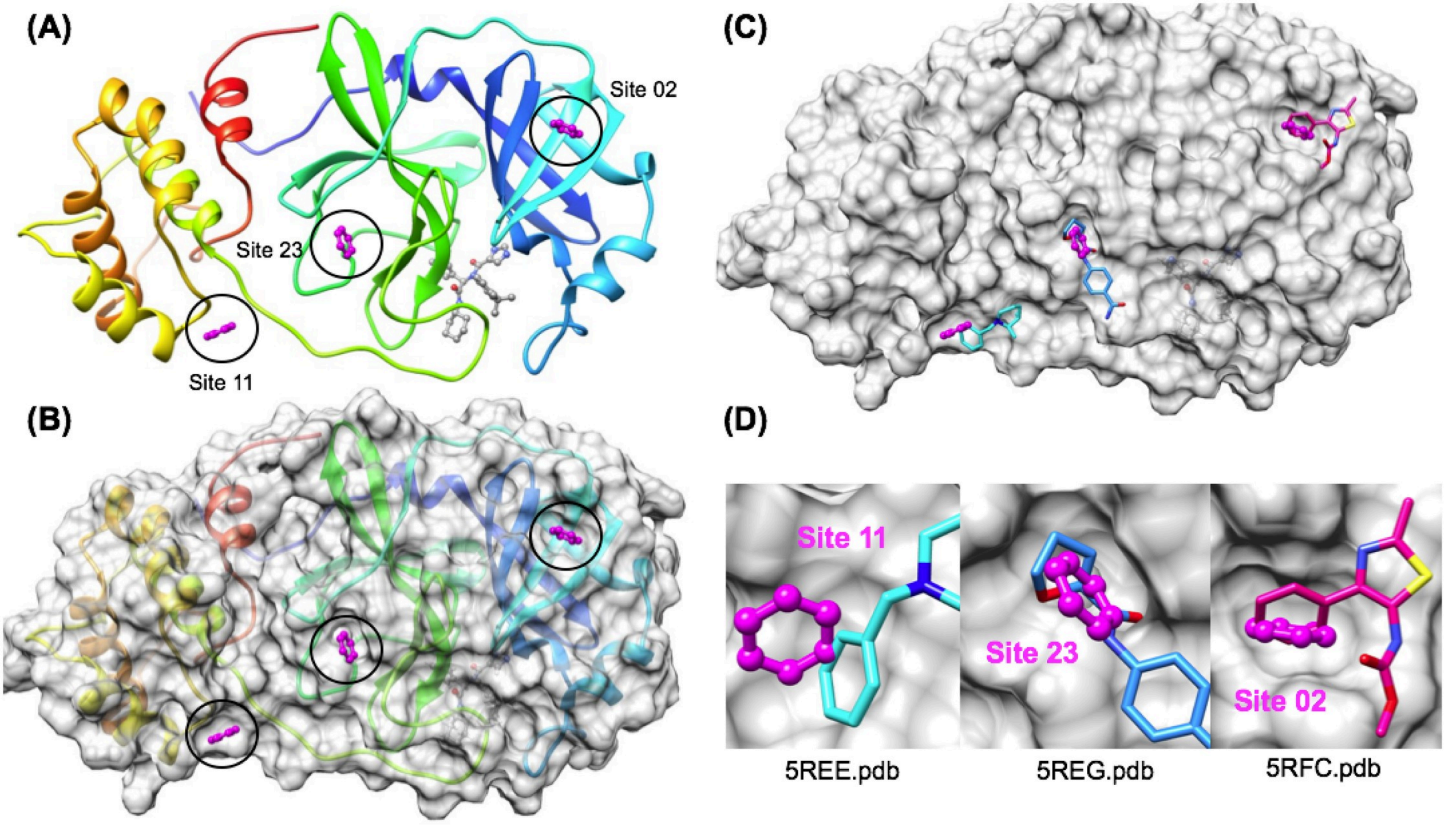
Experimental structures from fragment screening confirm other Nsp5 Mpro “minor” binding sites successfully predicted. The crystal structure of Nsp5 Mpro (6W63.pdb) is shown illustrating the reverse or “minor” binding surface using ribbon diagram with rainbow coloring (A) and is shown with a transparent gray surface in (B). Several crystal structures of other fragment ligands (5RFC.pdb, 5REE.pdb, 5REG.pdb) independently confirm the positions of the “minor” binding sites: Site 02, Site 11 and Site 23 pharmacophores shown in (C) and (D).

Next, we aimed to focus our analysis on other predicted binding sites that were beyond the well-known and obvious protease active site. In a comparison of our ranked pharmacophore binding sites with experimentally determined structures of the SARS-CoV-2 Nsp 5 Mpro from fragment screening, we were astonished to find that three other predicted “minor” binding sites were confirmed from independent co-crystal structure that we were not aware of (Date Accessed: August 11^th^, 2020). As shown on (Fig 7A) and (Fig 7B), all three of these allosteric fragment ligands bind on the surface opposite to the location of the enzyme active site. The most thermodynamically favorable of these sites was a member of the TOP5, Site 02 (LE = 0.34) which was also found to have a reasonable (RMSD = 1.14 Å) match over a six heavy atoms with true aromatic pharmacophore fragment ligands, such as the thiazole carbamate fragment ligand shown (5rfc.pdb) (Fig 7D) [70].

Of the “minor” binding sites that were verified by experimental structures, it is encouraging that the most favorable of these, Site 02 was also found to exhibit a reasonable pharmacophore match (RMSD = 1.14 Å) geometry. The next most favorable “minor” site, Site 08 (LE = 0.26) was a member of the TOP10, which was found to be a higher RMSD match (RMSD = 2.76 Å) with aromatic pharmacophore fragment ligands (5rgs.pdb 5ree.pdb, 5rec.pdb) such as the fragment ligand shown (5ree.pdb) (Fig 7D). Finally, in an interesting test of our assumptions with regards to the aromatic pharmacophore binding sites, we were able to find that one of the least favorable predicted sites, Site 23 (LE = 0.17) was also confirmed to have a fragment ligand bind in the location (5reg.pdb) [69]. This site was a very poor match of the aromatic group with a non-aromatic fragment group, but there was also local minor induced fit changes in the binding surface that would not be sufficiently accounted for in our current approach. In summary, while it is not that surprising that the overall pharmacophore mapping workflow was able to correctly identify the most important pharmacophores in the obvious major peptide binding active site, it is much more impressive and encouraging that three predicted allosteric fragment binding sites were confirmed by independent co-crystal structures. These observations follow recent reports that numerous protein targets exhibit one, two, three, and sometimes even four alternative small-molecule binding sites for fragment ligands [55, 56].

Following the identification and ranking by (ΔG_bind_), fragment screening was used to additionally characterize these sites. The results for screening the FRAG100 library are summarized in (Fig 5) and (S3 Fig in S1 File). The 3 sites comprising the Mpro active site have greater ligand efficiencies compared to the three other allosteric fragment binding sites over the top 10% or 20% of the sorted hit list. Of these allosteric sites, Site 02 was predicted to be the most favorable of these from pharmacophore mapping and was also shown in analysis of fragment screening to have the highest ligand efficiency of these where Site 02 (LE_max_ = 0.34) < Site08 (LE_max_ = 0.29) << Site 23 (LE_max_ = 0.20). Thus, the results from fragment screening reinforced the same trend that was observed from pharmacophore mapping, that Site02 had the greatest ligand efficiency of these experimentally confirmed allosteric fragment binding sites.

Turning our attention back around to the protease active site and the major P1, P2 and P3 sites, the results from fragment screening show that the site with the greatest ligand efficiency was the P2 sites (LE_max_ = 0.39), followed by the P1 site (LE_max_ = 0.35), and then the P3 site (LE_max_ = 0.33). Even though the P1 site was ranked to be slightly more favorable than the P2 site in the initial pharmacophore mapping results, this may have been due to it actually being a more exact match for an optimal group at this position. Fragment screening shows that the P2 site identified a greater number of more favorable (ΔG_bind_) and ligand efficient fragment hits than the P1 site. In summary, given that there was so much more structural information available the Nsp5 Mpro, our level of agreement with the existing data is encouraging and supports its use as a comparison benchmark against the other protein targets. The P1 site was selected as a reference site for screening with the larger 3,700 compound fragment screening library. We report this data below in the Nsp13 helicase section in comparison to screening the full fragment library against several Nsp13 helicase sites.

### Nsp16 2’-O methyltransferase

The Nsp16 is a 2’-O methyltransferase (MT) involved in 5’ cap maturation of viral RNA, which has been shown to be essential for viral replication in cell culture models for several viruses, including coronavirus strains [94, 95]. While 5’ cap formation is known to stabilize viral RNA and promotes effective translation, 2’-O methylation of the 5’ cap of viral RNA was also recently shown to play a specific role in evading innate immune system antiviral responses involving type I interferon signaling [94]. A recent crystal structure of the SARS-CoV-2 Nsp16 in complex with the broad-spectrum MTase inhibitor sinefungin was used for our structural studies [74, 96]. Sinefungin is an agent with some broad-spectrum antiviral activity. Interestingly, while sinefungin is a very potent inhibitor of some viral MTase enzymes such as poxvirus vaccinia virus (75 nM) and Newcastle disease virus (150 nM) [97] it is a much weaker inhibitor of coronaviruses [95], but represents a reasonable starting point for structure-based-design of more potent SARS-CoV-2 Nsp16 2’-O MTase inhibitors [96].

Similar to the Nsp5 Mprot, the Nsp16 2’-O MT is a relatively smaller protein (296 residues) compared to the larger targets and the TOP25 binding sites were identified. The analysis was performed using the recent structure of the Nsp16 / Nsp10 homodimer bound in complex with sinefungin [74, 96]. The structure of Nsp16 was analyzed removing the structure of the smaller binding protein Nsp10 and therefore several of our most favorable TOP25 sites were found to also map to Nsp16 / Nsp10 protein-protein interaction (PPI) sites.

**Fig 8:**
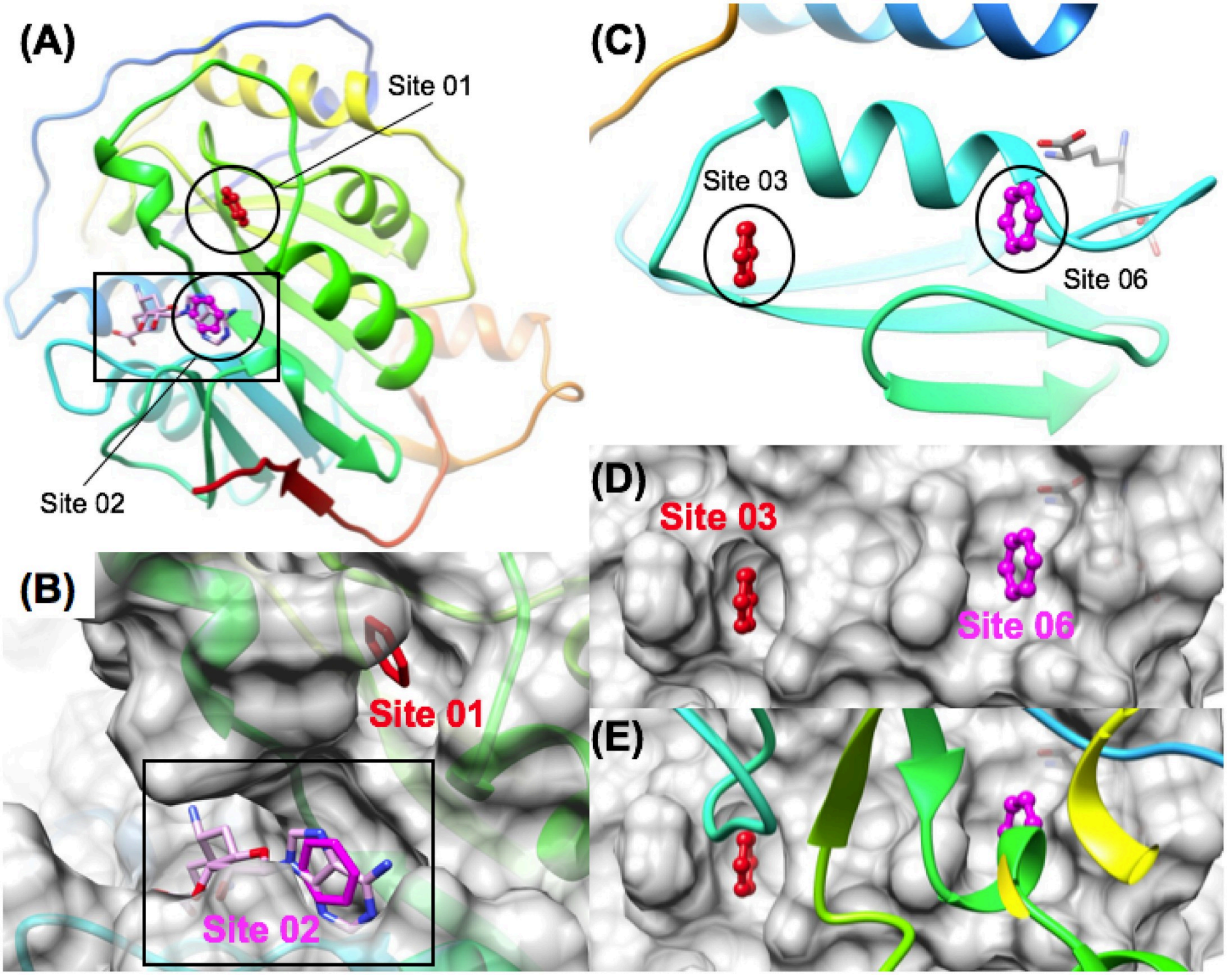
Pharmacophore mapping successfully identifies the Nsp16 sinefungin binding site and other PPI sites. The most favorable two sites identified are shown on a ribbon diagram in (A) and on a surface representation in (B) where Site 01 (red) was the most favorable site identified that was proximal to Site 02 (magenta), which superimposes with the most favorable pharmacophore site in the bound sinefungin structure. Two protein-protein interaction (PPI) sites on the Nsp16 binding surface were identified as favorable aromatic pharmacophore binding sites shown in (C) and (D). Site 03 (red) and Site 06 (magenta) represent Nsp16 / Nsp10 heterodimer interaction sites where the Nsp10 ribbon is shown (E) in rainbow binding to the complementary Nsp16 surface shown in gray.

When the TOP25 sites were ranked by (ΔG_bind_), it was not surprising that a specific substructure of sinefungin was found to be one of the most favorable sites within the TOP5. The sinefungin adenine group pharmacophore Site 02 (LE = 0.40) was found to be the 2^nd^ most favorable overall site of the TOP25 and the most favorable site that maps to the structure of sinefungin. Although this result is entirely expected, it remains in sharp comparison to the sinefungin ribose pharmacophore position identified as Site 25 (LE = 0.15) which is much less favorable. Also, ribose is a poor replacement of a flat aromatic benzene pharmacophore, so it is also expected to not be very favorable. Interestingly, the most favorable overall site on Nsp 16 was, Site 01 (LE = 0.42) in a hydrophobic pocket that is directly adjacent to the sinefungin binding site formed by residues (res: D6931, K6933, F6947, F6948, K6944). This site is illustrated in (Fig 8A) as the close proximity of Site 01 (LE = 0.42) and Site 02 (LE = 0.40) is interesting as it is on the order of 3–4 bond lengths and a short distance of 6.9 Å between the two closest benzene pharmacophore heavy atoms. Inhibitors with moieties that bind to this Site 01 hydrophobic pocket may offer selectivity advantages according to a structural alignment with human 2’O MTase enzymes (4N48.pdb), which shows that human enzymes have entirely different structural features at the equivalent position [98]. Since sinefungin derivatives are potential broad-spectrum agents with lots of potential uses [99–101], this could be of significant interest as it may offer an avenue for sinefungin derivatives (or replacements) with improved activity and ADME profiles. Sinefungin has been shown to be nephrotoxic following IV injection [102], so derivatives more optimized for SARS-CoV-2 may be able to improve potency, selectivity and toxicity profiles.

Turning our attention to locations other than the major sinefungin binding site, two PPI sites on the Nsp16 binding surface were identified shown in (Fig 8B) that represent Nsp16 / Nsp10 heterodimer interface PPI sites that were ranked in the TOP6 by (ΔG_bind_). The most favorable of these were Site 03 (LE = 0.32) and Site 06 (LE = 0.28), in comparison to two other less favorable PPI sites Site 12 (LE = 0.25) and Site 18 (LE = 0.23). So, as might be expected, specific PPI sites are more favorable than other so called “hot-spots.” However, all of these Nsp16 / Nsp10 dimer interface PPI sites were less favorable than the major small-molecule co-factor binding site, the sinefungin adenine group pharmacophore Site 02 (LE = 0.40), as expected. Targeting the Nsp10:Nsp16 PPI sites have been proposed for SARS-CoV-2 discovery [103] and Nsp10 derived peptide inhibitors of the Nsp10:Nsp16 interaction have been previously demonstrated to be effective to inhibit SARS-CoV Nsp16 MTase activity [104].

Subsequent fragment screening into these the most favorable identified sites on Nsp16 also demonstrated that the two most favorable overall sites, Site 01 and Site 02 both had much higher calculated ligand efficiencies compared to the most favorable PPI site 03 as shown in (Fig 5), and (S3 Fig in S1 File). Fragment screening shows that the site with the greatest ligand efficiency was sinefungin adenine group pharmacophore Site 02 (LE_max_ = 0.48), followed by the adjacent Site 01 (LE_max_ = 0.44), and then followed by the two most favorable PPI sites, Site 03 (LE_max_ = 0.33) and Site 06 (LE_max_ = 0.27) respectively. Thus, even though Site 01 was ranked to be slightly more favorable than the Site 02 in the pharmacophore mapping results, fragment screening was able to show that the Site 02 sinefungin adenine group pharmacophore exhibited the greatest ligand efficiency of any of the sites on Nsp16 as expected.

### Nsp12 RNA-dependent RNA polymerase (RdRp)

Computational analysis was performed using the recent structure of the Nsp12 RdRp in complex with Nsp7, Nsp8 and Remdesivir [72]. The structure of Nsp12 was analyzed removing the structures of the smaller binding proteins Nsp7 and Nsp8. Therefore, several of our most favorable TOP50 sites were found to also map to Nsp7 or Nsp8 protein-protein interaction (PPI) sites on the surface of Nsp12 [72]. When the TOP50 sites were ranked by (ΔG_bind_), it was somewhat surprising that the top-ranked site would be a PPI site. Site 01 (LE = 0.48) is a very favorable PPI site (for Nsp8). Overall, 4 other binding sites were mapped to Nsp7 and Nsp8 PPIs respectively. Of the four Nsp7 PPI sites, Site 11 (LE = 0.28) was the most favorable, followed by Site 18 (LE = 0.25), Site 28 (LE = 0.22), and Site 45 (LE = 0.17) respectively. In comparison the four Nsp8 PPI sites were more favorable where Site 01 (LE = 0.48) was the most favorable, followed by Site 08 (LE = 0.30), next by closely adjacent Site 20 (LE = 0.25) and Site 31 (LE = 0.22) respectively.

As RdRp is a much larger protein with a more complex multi-domain structure, the results were also compared with the same protocol using reference “knowledge-based” pharmacophores as input. A recent study of RdRp used an interaction fingerprint method to study inhibitor binding modes and identify common inhibitor binding motifs for SARS-CoV-2 RdRp [105]. Several representative reference structures (7bv2.pdb) (5f3z.pdb) (3cwj.pdb) from this analysis were used to derive putative benzene pharmacophore positions from these representative viral RdRp inhibitor complexes [72, 106, 107]. Six reference pharmacophore positions where used as input for the workflow, including substructures of the reference inhibitor remdesivir (7bv2.pdb) [72]. When the results were ranked by (ΔG_bind_), it was not surprising that the two most favorable reference pharmacophore positions were found to map to the nucleotide binding site. Interestingly, the most favorable site superimposed with the ribose group (LE = 0.32) and the pyrrolotriazin-4-amine group (LE = 0.22) of the bound reference inhibitor remdesivir (7bv2.pdb). Other virtual screening studies have identified natural products and nucleoside analogs that bind favorably to this site [108]. The third most favorable reference site (LE = 0.21) was derived from a structure of HCV Ns5b in complex with a non-nucleotide pyridazinone inhibitor [107]. In comparing results for these reference knowledge-based pharmacophores to the ranked TOP50 binding sites, the most favorable pharmacophore position from the reference inhibitor remdesivir (LE = 0.32), would have ranked as 5^th^ among the TOP50 binding sites. The next most favorable non-nucleotide binding site (LE = 0.21) would have ranked as 33^rd^ among the TOP50. This comparison highlights the predicted thermodynamic favorability of several of the top-ranked sites. Site 01 (LE = 0.48) is a PPI site (for Nsp8) and is predicted by our analysis to be able to bind fragment ligands as favorably as the remdesivir binding site.

**Fig 9:**
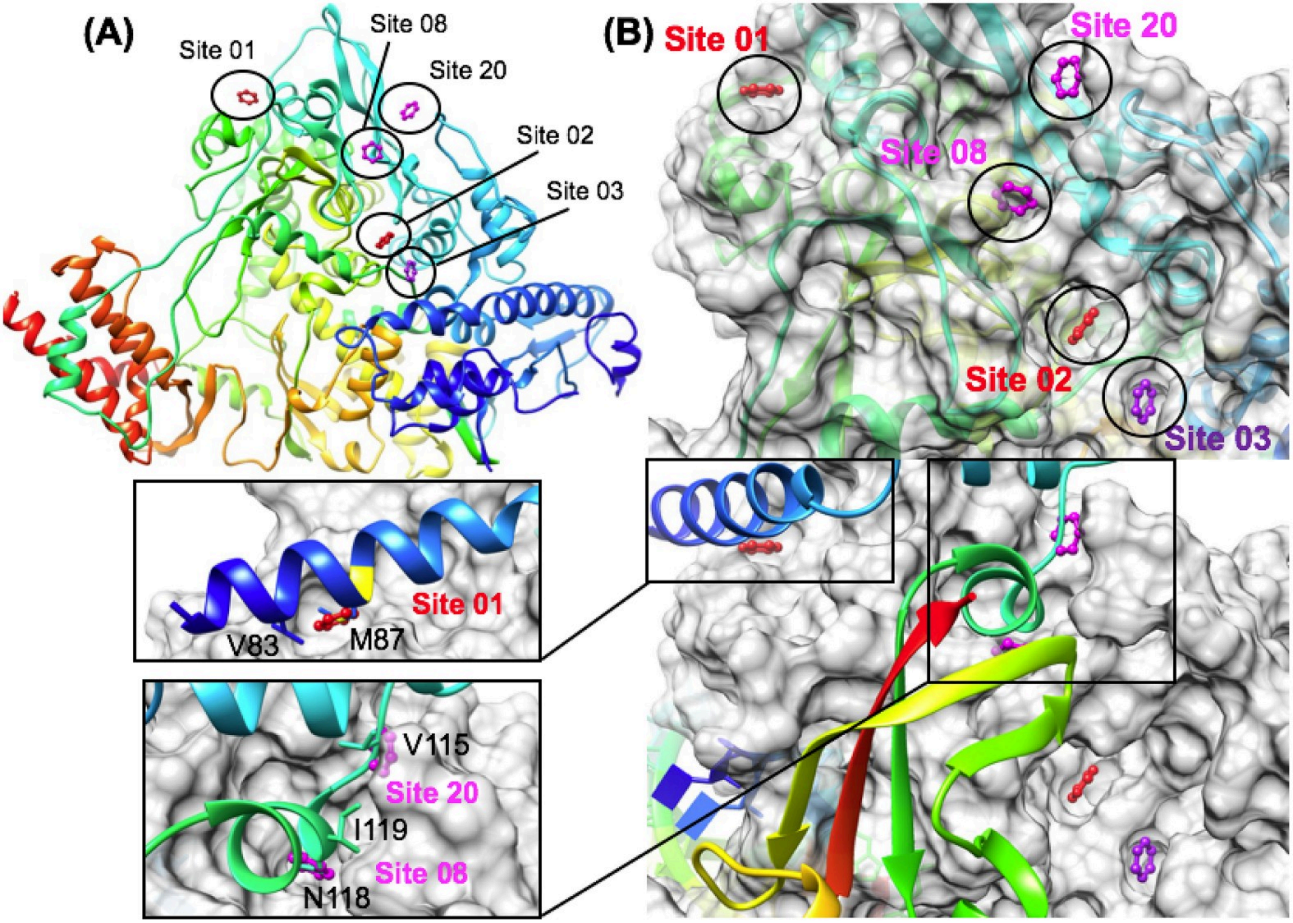
Favorable aromatic pharmacophore binding sites identified on the Nsp12 RdRp. Favorable sites are shown in red or magenta on the rainbow ribbon structure shown in (A) and on the gray binding surface shown in (B). Several protein-protein interaction (PPI) sites on the Nsp12 binding surface were identified as favorable aromatic pharmacophore binding sites shown in (B). Site 01 (red), Site 08 and Site 20 (magenta) represent Nsp12 / Nsp8 heterodimer interaction sites where the Nsp8 ribbon is shown (B) in rainbow binding to the complementary Nsp12 surface shown in gray. Zoom in surface views are shown to illustrate corresponding Nsp8 hydrophobic residues side chains that participate in PPI on the Nsp12 surface.

Within the ranked TOP50 binding sites, the next two most favorable sites that were not associated with an Nsp7 or Nsp8 PPI were Site 02 (LE = 0.35) and Site 03 (LE = 0.34). These two sites are very close to each other in proximity and are illustrated in (Fig 9). As we finalized our manuscript for submission, the entirely novel CryoEM structure of the Nsp12 RdRp transcription replication complex [61] became known to us (Date Accessed: Aug 27th, 2020), elucidating never before characterized interactions between Nsp12 and multiple copies of the Nsp13 [61]. In our comparison of the structure utilized for the solvent mapping studies (7bv2.pdb) [70] and the new replication complex (6xez.pdb) [61], when the structures of the Nsp12 RdRp are superimposed or matched by the backbone C_α_ carbon, the protein surface for these two most favorable binding sites Site 02 (LE = 0.35) and Site 03 (LE = 0.34) on Nsp12 exhibit very minimal deviation in structure between the two independent experimental structure determinations of Nsp12. Thus, these sites exhibit minimal structural deviation between available independent structural snapshots of the active complex and are thermodynamically favorable for small-molecule binding. These two binding sites are seemingly bisected by the segment of (res:459–465) in the finger domain which spans between a N-terminal 3_10_-helix (res: 453–458) and a C-terminal α-helix (res: 466–480). Site 02 (LE = 0.35) composed from residues (R349, P461, P667) is formed by the previously mentioned (res:459–465) of the finger domain and (res:312–350) on the interface domain. In close proximity, Site 03 (LE = 0.34) is composed from residues (L172, L460, P461), where it is formed by similar residues (res:459–465) of the finger domain and the interface with several residues (L172, T246, L247, R249) from the N-terminal NiRAN domain. To the best of our knowledge, the exact role of these binding sites is currently unknown. Although it is possible that this could be a binding site for another protein, we have currently not found any evidence to support this from mutational studies or PPI mapping studies to date.

Following identification of these various favorable sites, subsequent fragment screening also demonstrated very favorable ligand efficiencies for four sites on Nsp12 RdRp: Site 01 (LE_max_ = 0.34), Site 02 (LE_max_ = 0.37), Site 03 (LE_max_ = 0.39), Site 08 (LE_max_ = 0.35). In comparison to another protein, all four of these RdRp sites exhibit greater than or equal to LE_max_ to the reference (P1) Nsp5 Mpro site. The most favorable Nsp8 PPI site from pharmacophore mapping, Site 01 (LE_max_ = 0.34) was found to have very favorable ligand efficiencies compared to another less favorable Nsp8 PPI site, Site 20 (LE_max_ = 0.27).

### Nsp13 helicase

**Fig 10:**
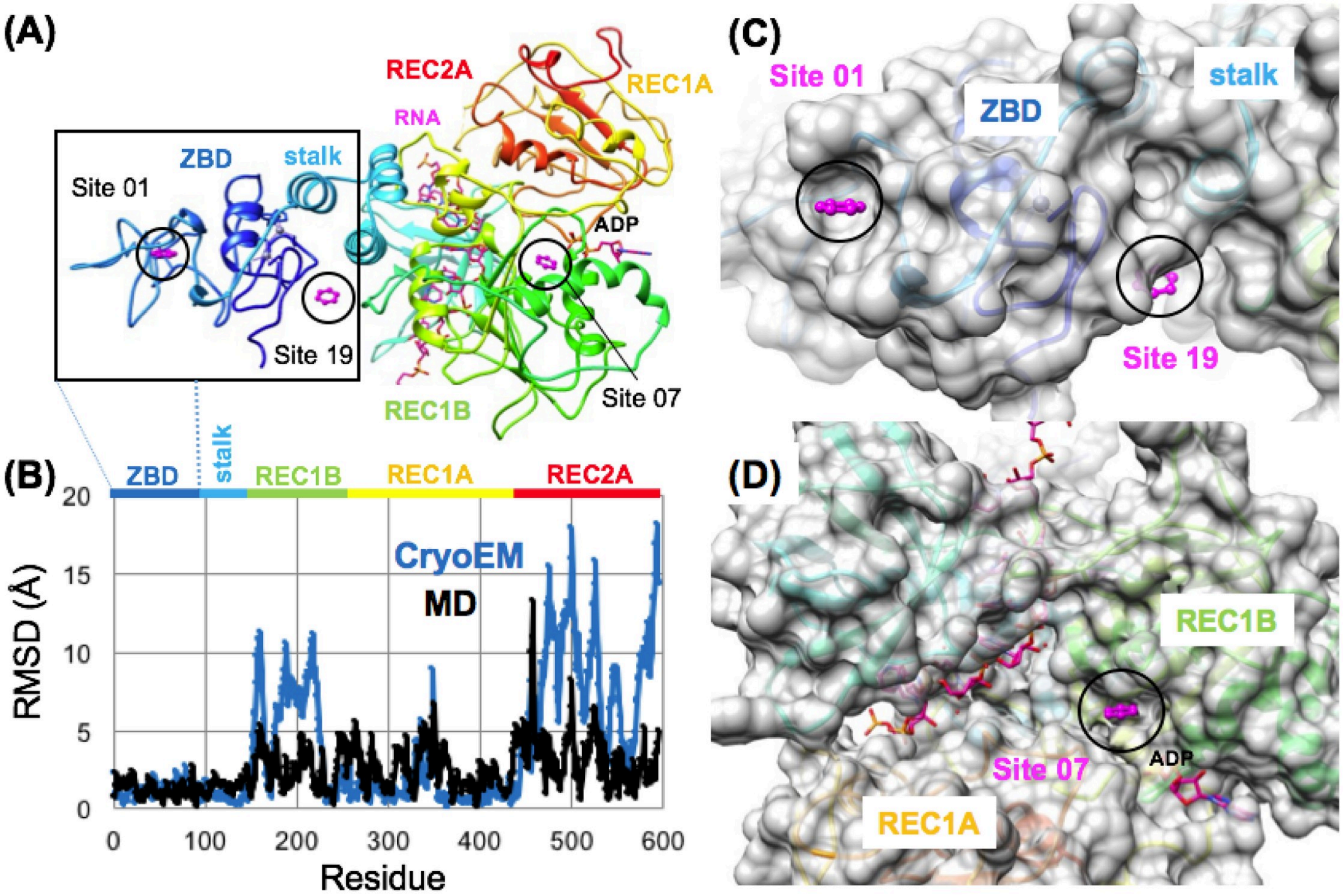
Domain structure and favorable aromatic pharmacophore sites on the Nsp13 helicase. Nsp13 structure is shown in a rainbow ribbon diagram in (A) where the N-terminal zinc-binding-domain (ZBD) is colored blue and the C-terminal REC2A domain is colored red. Favorable binding sites Site 01, Site 19 and Site 07 are highlighted in magenta and labeled. The location of RNA and ADP binding are modeled onto the structure via a structural alignment of another eukaryotic helicase (2xzl.pdb) bound to RNA and ADP. In (B) the root-mean-squared-deviation (RMSD) calculated between two recent Nsp13 helicase structures captured in different conformational states in a CryoEM structure of a complete SARS-CoV-2 replicase complex is shown in (blue). A similar RMSD as a function of residue plot is shown for 500 ps of standard molecular dynamics (MD) simulation of the (6jyt.pdb) structure at 310 K in all-atom solvent (black). Favorable binding sites Site 01, Site 19 and Site 07 are highlighted in magenta and shown bound to the Nsp13 helicase surface in (C) and (D) where Site 01 is the most favorable site on the entire Nsp13 helicase found on the N-terminal ZBD (C), where Site 07 was the most favorable site identified in the vicinity of the ATPase active site (D).

The helicase functions to separate double stranded nucleic acid (dsNA) by utilizing energy from nucleoside triphosphate NTP during translocation on single stranded nucleic acids (ssNA) necessary in prokaryotes and eukaryotes for genome replication and recombination. The helicase has a shape of a triangular pyramid (Fig 10A) that is divided into five sections, a Zinc Binding domain (ZBD) which is attached to two REC-A domains (REC-1A and REC-2A) and a REC-1B domain via a stalk domain (Fig 10B) [58, 109]. Relevant to drug development, it should be noted that the ZBD, stalk, and 1B domains of the helicase are unique to nidovirales, which presents an opportunity for more selective drug targeting [61]. The N-terminal ZBD of the helicase is one of the most conserved domains across the order nidovirales [110]. The catalytic core of the helicase is formed by the two RecA-like domains, the 1A and 2A, where the B19-B20 loop on the A1 domain is directly involved with RNA unwinding [57, 110]. While the exact role of the ZBD is unclear, we do know that it is crucial in the viability of nidovirales, a study on the ZBD of the nidovirales Equine Arteritis virus Nsp10 showed that alterations to Cys/His residues that coordinate zinc binding, rendered the virus nonviable [111].

When the TOP50 sites were ranked by (ΔG_bind_), several of the top-ranked sites were found on the N-terminal ZBD domain (Fig 10C) rather than in the vicinity of the ATPase active site (Fig 10D), or other RNA-binding areas. Interestingly, the TOP3 ranked sites, Site 01 (LE = 0.31), Site 02 (LE = 0.25), Site 03 (LE = 0.25) were all found on the N-terminal ZBD domain. Site 01 remained the most favorable of these three sites following subsequent fragment screening analysis and is shown in (Fig 10C). Following ranking by (ΔG_bind_) two other N-terminal ZBD sites were also identified that were specifically adjacent to each other, the more favorable Site 06 (LE = 0.24) and the less favorable Site 19 (LE = 0.21) shown in (Fig 10C). So, all five of these predicted N-terminal ZBD domain sites were calculated to be much more favorable than Site 32 (LE = 0.19), which was an aromatic pharmacophore match to the ADP ribose binding site at the ATPase catalytic site. ADP and RNA binding locations modeled in (Fig 10) are shown in magenta. These models were derived from a structure-based alignment of our SARS-CoV helicase structure (6jyt.pdb) and eukaryotic Upf1 helicase (2xzl.pdb) [112]. Beyond the most favorable sites predicted on the N-terminal ZBD domain, the next most favorable Site 07 (LE = 0.24) was proximal to the ATP binding site shown in (Fig 10D). The other most favorable sites found in the RNA binding channel were Site 13 (LE = 0.23) and the closely adjacent Site 17 (LE = 0.22). Other favorable sites identified in the RNA binding channel include Site 18 (LE = 0.21) and Site 23 (LE = 0.21).

As the helicase is a large multi-domain protein, the results were also compared with the same protocol using reference “knowledge-based” pharmacophores. A total of 16 reference pharmacophore positions for RNA and ATP binding sites were derived from a structural alignment with eukaryotic Upf1 helicase (2xzl.pdb) [112]. An additional 11 reference pharmacophore positions were derived in a similar way using several representative reference structures: (4b71.pdb) [55], (5fps.pdb), (5fpt.pdb), (5fpy.pdb) [56] (2zjo.pdb) [113] (3rvb.pdb) [114] of hepatitis C virus Ns3 helicase inhibitor complexes. When the results were ranked by (ΔG_bind_), the most favorable reference pharmacophore positions for RNA and ATP binding were found to map to the ATP binding site. The majority of the RNA binding sites were rather unfavorable in comparison. For the adenosine binding site, the reference adenine site (LE = 0.22) was found to be more favorable for an aromatic pharmacophore than the ribose site (LE = 0.20) as might be expected. When the reference pharmacophores from helicase inhibitors were ranked by (ΔG_bind_), the most favorable site (LE = 0.23) was derived from a reference fragment ligand (5fps.pdb) and other reference inhibitors were also found to share ligand density at this position (2zjo.pdb) [113]. This lowest energy “knowledge-based” pharmacophore was also found to be a short distance (< 1.5 Å) from the previously identified Site 07 (LE = 0.24) from the TOP50.

In comparing results for these knowledge-based pharmacophore to the ranked TOP50 binding sites, the most favorable pharmacophore position from the reference inhibitors (LE = 0.23), would have ranked as 11^th^ among the TOP50 binding sites and a very similar site was previously identified and ranked 7^th^. This comparison highlights the predicted thermodynamic favorability of several of the top-ranked allosteric sites that would not have been identified from only a knowledge-based approach. Site 01 (LE = 0.31) on the ZBD is predicted by our analysis to be able to bind fragment ligands as favorably or more favorably than Site 07 (LE = 0.24) or any of the knowledge-based reference sites.

Following pharmacophore mapping and ranking of these sites, subsequent fragment screening was performed with the smaller fragment library [FRAG100]. These results in (Fig 5D) show that the N-terminal ZBD Site 01 (LE_MAX_ = 0.37) was the most favorable of all the helicase sites and also exhibited the greatest calculated ligand efficiencies. The next most favorable site (LE_MAX_ = 0.31) was Site 07 which was the most favorable site identified and is proximal to the ATP binding site. Site 07 was also a short distance (< 1.5 Å) from the most favorable knowledge-based pharmacophore (LE = 0.23), where both sites share close interactions with residue K288 which is required for catalytic NTPase activity [110, 115] and within (< 3.5 Å) of the catalytic Mg^2+^ binding site according to the structural alignment with eukaryotic Upf1 helicase (2xzl.pdb) [112]. Thus, our analysis suggests that Site 07 is the most favorable reference pharmacophore site identified for virtual screening for direct inhibitors of NTPase activity.

**Fig 11:**
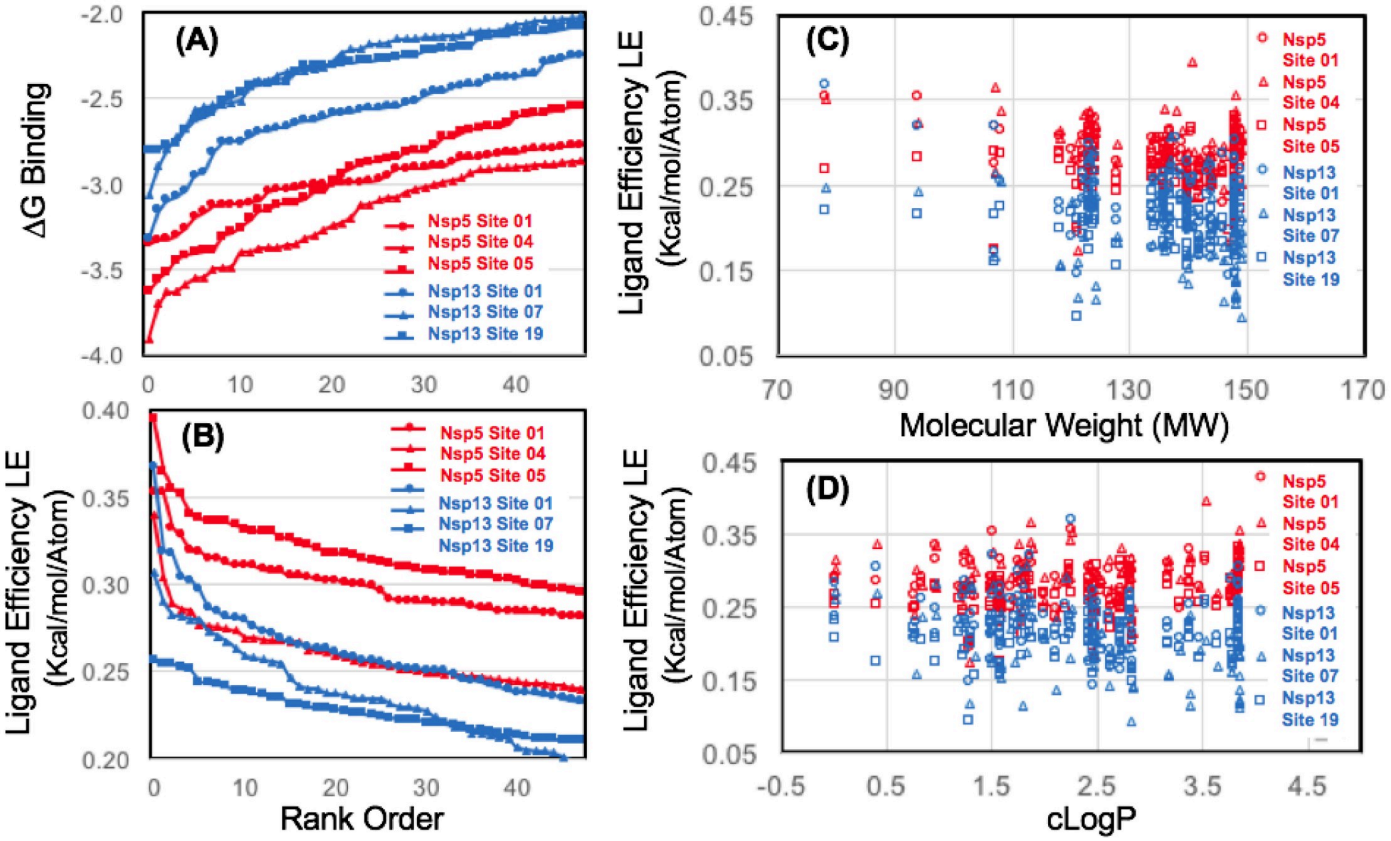
Ligand efficiency for several favorable binding sites on Nsp5 Mpro and Nsp13 helicase from computational fragment screening. Data is shown for ΔG_bind_ (A) and ligand efficiency (B) comparing numerous sites using the FRAG100 library as a benchmark. Ligand efficiency data is also shown as a function of fragment molecular weight (C) and calculated LogP (D).

From these results, compared to all of the other helicase sites the next most favorable was a ZBD-stalk interface allosteric Site 19 (LE_MAX_ = 0.26) shown in (Fig 5D). This was also interesting with regards to the workflow computational methodology, as Site 19 was ranked much lower in both previous pharmacophore mapping steps, both the mapping N contacts criteria, and in calculated (ΔG_bind_), but was found to have reasonable ligand efficiency with fragment screening derivatives identified from the library. Shown in (Fig 11) is a comparison of fragment screening data for the three most favorable sites on the Nsp13 helicase (as identified from this step) compared to the three major P1, P2, P3 binding sites of the Nsp5 Mpro. As can be seen in (Fig 11), the most favorable site on the Nsp13 helicase, Nsp13 Site 01 is still not as favorable as the three major binding sites of the Nsp5 Mpro, by ΔG_bind_. However the ligand efficiencies for Nsp13 Site 01 are on the order of the P3 site (Nsp5 site 04), but not as favorable as the other two P1 (Nsp5 Site 01) and P2 sites (Nsp5 Site 05).

**Fig 12:**
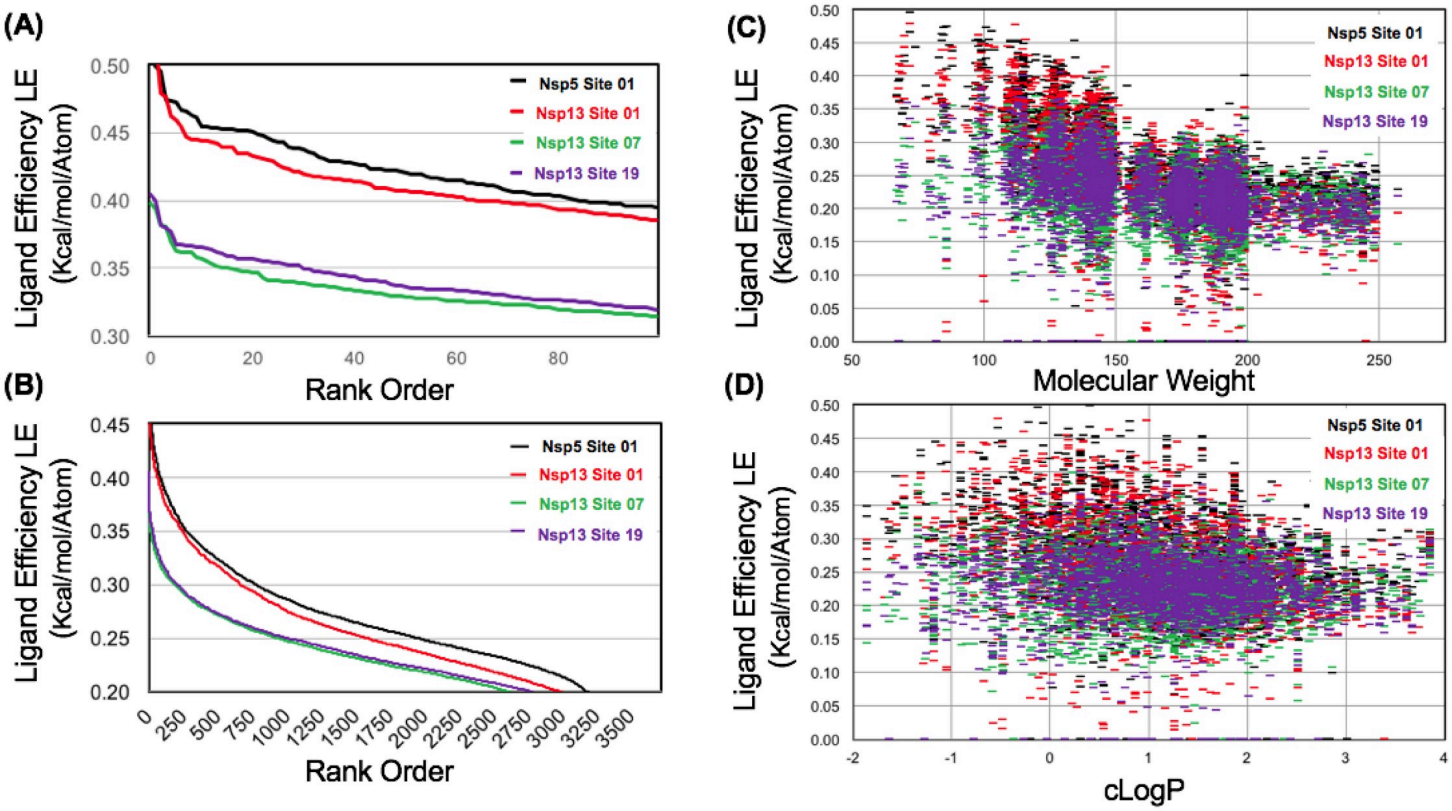
Ligand efficiency for favorable binding sites on Nsp5 Mpro and Nsp13 helicase from computational fragment screening. Data is shown for ligand efficiency (A) and (B) comparing several sites using the entire FRAG3700 library. Ligand efficiency data is also shown as a function of fragment molecular weight (C) and calculated LogP (D).

The larger fragment library (3,700 cmp) was screened for these three most favorable sites on the helicase and the results were compared to the “reference” P1 aromatic pharmacophore site on Nsp5 Mpro Site 01 (LE_MAX_ = 0.52). Shown in (Fig 12), following screening with the diverse heterocycle replacement library, none of the Nsp13 helicase sites were able to achieve calculated ligand efficiencies greater than the P1 protease site. Of these helicase sites, the N-terminal ZBD domain Site 01 (LE_MAX_ = 0.50) was found to have the greatest ligand efficiency over the entire database. The ZBD-stalk domain interface Site 19 (LE_MAX_ = 0.40) was actually found to have a slightly greater ligand efficiency over the entire database than Site 07 (LE_MAX_ = 0.39) which was the most favorable site identified proximal to the ATPase active site. A conclusion from our screening data analysis is that at least two allosteric ZBD domain binding sites may be at least as favorable, or more favorable compared to the ATPase active site ATP binding site.

**Fig 13:**
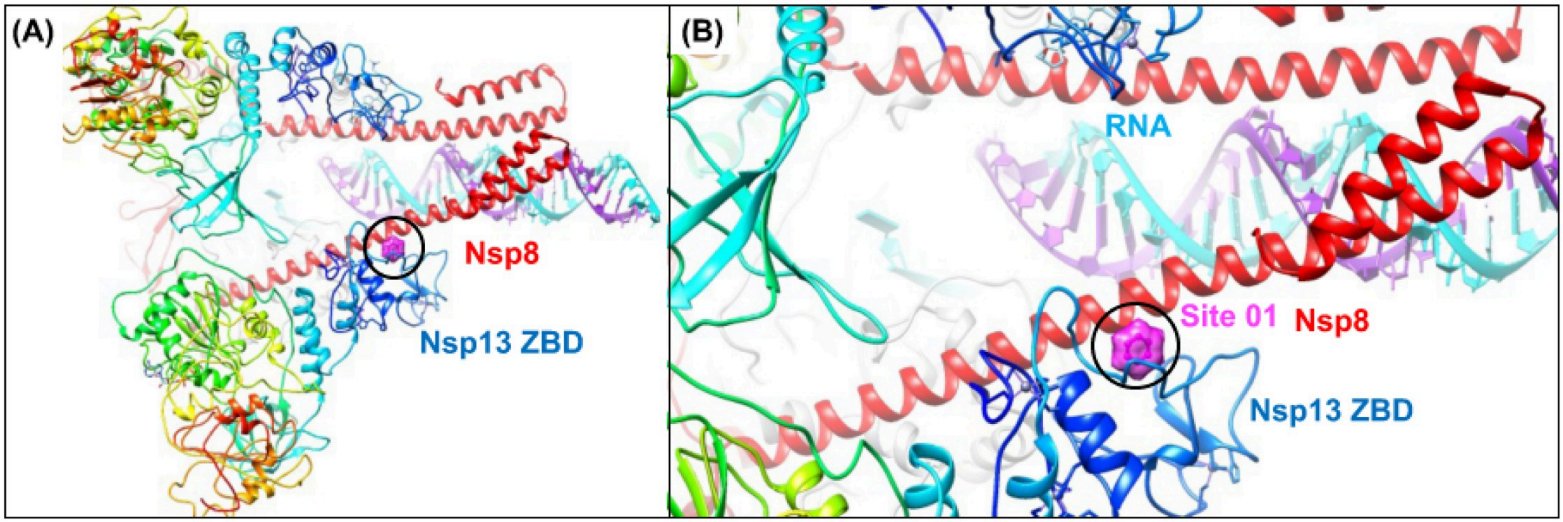
Structure of the Nsp12: Nsp13 replication transcription complex reveals that the Nsp13 zinc-binding-domain (ZBD) mediates protein-protein interactions. (A) A ribbon diagram of the structure of the complex (6xez.pdb) [61] shows how two subunits of the helicase form protein-protein interactions with both Nsp8 (red) and Nsp12 in the active complex through interactions with the helicase ZBD domain (blue). (B) A zoom in view shows that the previously predicted Site 01 on the ZBD (magenta) is proximal (within 5–6 Å) to the Nsp8:Nsp13 protein-protein interaction site in the replication complex.

Prior to the release of the cryoEM structure of the replication transcription complex of Nsp12:13 (discussed in the next section), based on these fragment screening results, we had hypothesized that small molecules binding to these N-terminal ZBD allosteric sites may interfere with Nsp13 helicase function either inhibiting (1) conformational change of the ZBD domain recently identified to be key in the allosteric helicase mechanism or (2) protein-protein interactions with Nsp8 forming the replication complex [110]. The release of the cryoEM structure of the replication transcription complex of Nsp12:13 provided previously unavailable structural clarity to that issue. The complex demonstrates for the first time how a patch of residues of the Nsp13’s ZBD participate in protein-protein interactions with both Nsp8 and Nsp12 in the active complex. This new structure shows that the predicted Site 01 on the ZBD is proximal (within 5–6 Å) to the newly revealed Nsp8:Nsp13 protein-protein interaction site in the replication complex (Fig 13). Site 01 is formed by reasonably conserved residues F81 and L83 and highly conserved residues F90 and G91, where F81, L83, and F90 also form key PPI contacts with Nsp8 (res:59–67). Based on our fragment screening results and the structural characterization of this functional PPI site, it appears that targeting this ZBD protein-protein interaction site with small-molecules may be sufficient to disrupt complex formation. In addition, this new cryoEM structure also experimentally confirmed that weak affinity small-molecule binding is possible at the predicted Site 01 on the ZBD of the Nsp13 helicase. In retrospect, the cryoEM structure of the replication transcription complex became publicly available to the public on the pdb database (Aug 2020), but we were unaware of the structure until (Access Date: Aug 27^th^, 2020).

### Small-molecule binding site on the Nsp 13 zinc binding domain (ZBD)

**Fig 14:**
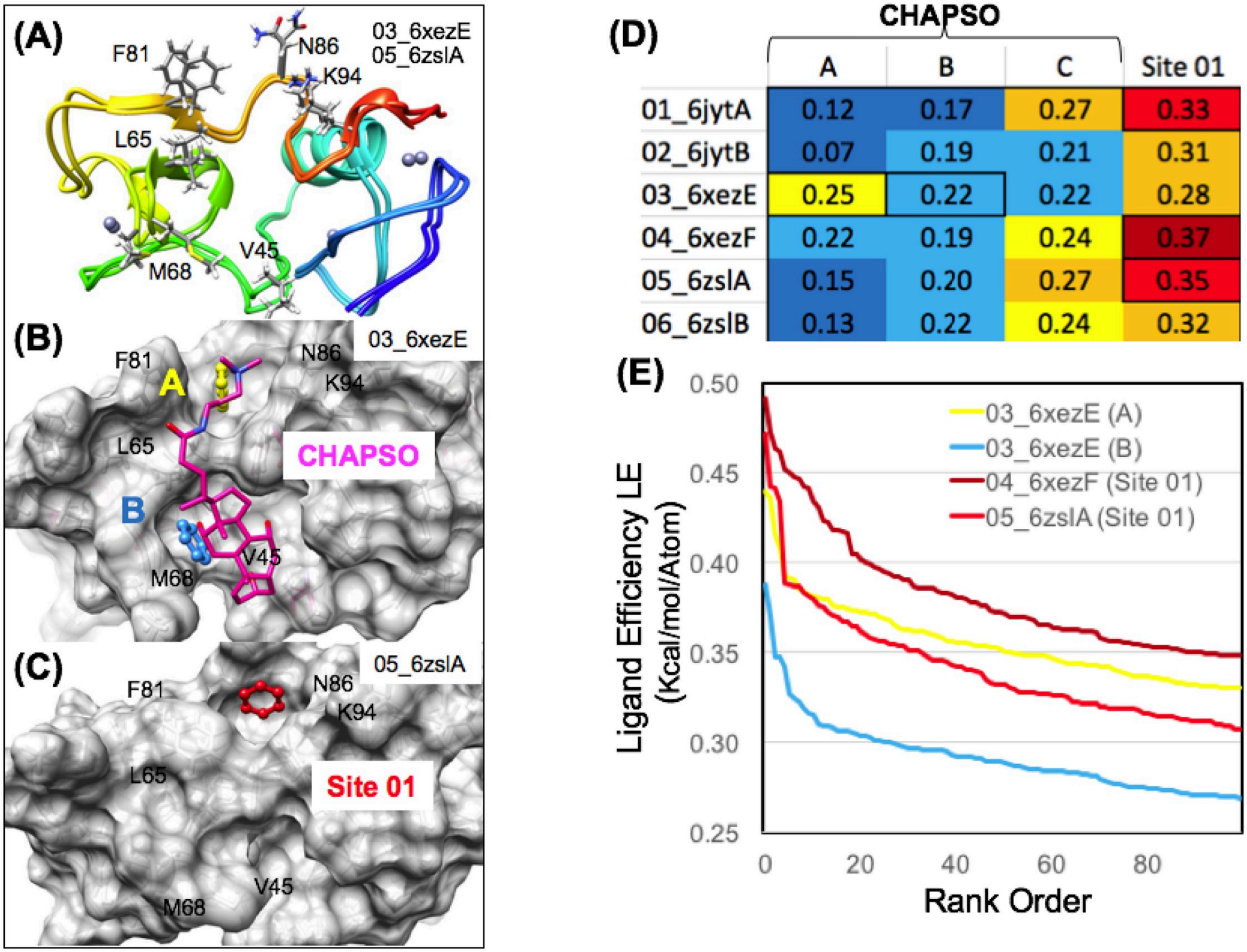
Nsp13 helicase allosteric zinc-binding-domain (ZBD) small-molecule binding site. The ZBD structures of the flexible residues forming Site 01 are shown on a ribbon diagram in (A) and the residues involved in Site 01 and CHAPSO binding are labeled. A surface diagram (B) shows the complementary surface of the ZBD (6xezE.pdb) conformational state captured binding to CHAPSO. The most favorable aromatic pharmacophore groups identified on this surface were labeled “A” (yellow) and “B” (blue). A surface diagram is shown in (C) showing the complementary surface of the ZBD on the new crystal structure (6zslA.pdb) where Site 01 is also found to be extremely favorable, ligand efficient (LE = 0.35) and clashing with the CHAPSO binding site. Cross-docking analysis calculating ligand efficiencies for these pharmacophore sites (D) show that previously identified Site 01 is the most favorable of all of these at the CHAPSO binding site. Site 01 exhibits the highest ligand efficiency in three independent structures [01_6jytA, 04_6xezF, and 05_6zslA]. Ligand efficiency from computational fragment screening (FRAG1000) shown in (E) demonstrates that Site 01 is more favorable for ligand binding in the 04_6xezF and 05_6zslA ZBD conformational states, compared to the CHAPSO 03_6xezE “B” (blue) pharmacophore site.

The recent 3.5 Å resolution cryoEM structure (6xez.pdb) of the replication transcription complex of Nsp12 RdRp in complex with Nsp7, Nsp8 and Nsp13 shows for the first time how the Nsp12 RdRp couples with multiple copies of the Nsp13 helicase [61]. While the majority of our studies had been performed using the structure (6jyt.pdb) [57], this new cryoEM structure (6xez.pdb) and a new 1.94 Å crystal structure (6zsl.pdb) of a Nsp13 helicase dimer complex offers unprecedented opportunities to compare these new independent snapshot structures of the Nsp13 helicase [61, 62]. These structures result in six total independent monomeric structures of the full-length Nsp13 proteins. In the 3.5 Å resolution cryoEM structure (6xez.pdb) of the replicase complex, the copy of Nsp13 that is chain E is found to have a molecule of the detergent CHAPSO bound on the Nsp13 N-terminal ZBD [61]. This CHAPSO binding sites overlapped with the single most favorable site on the Nsp13 helicase identified in our previous pharmacophore mapping and fragment screening, the ZBD Site 01 (LE_MAX_ = 0.50) as shown on (Fig 14). Interestingly, in a comparison of the 6 independent structures of the Nsp13 helicase, the ZBD remains as a relatively rigid domain, compared to other domains that exhibit major deviations such as REC1B and REC2A as shown on (Fig 10B) depending on the specific conformational state of Nsp13 in the replication transcription complex.

However, despite the fact that the Nsp13 N-terminal ZBD domain remains relatively rigid (Cα RMSD < 1.0 Å) between multiple independent structures and in short MD simulations in explicit solvent, the independent structures of the SARS-CoV-2 structures show that small side-chain mediated conformational changes in the binding site are observed between the different structures (Fig 14A). Interestingly, this analysis illustrates that the binding of the very inefficient detergent ligand CHAPSO induced minor side-chain conformational changes in the vicinity of this binding site. For a more thorough and exhaustive analysis of these conformational changes and effects on fragment ligand binding, the six independent crystal structure conformations were utilized for cross-docking analysis (6jytA, 6jytB, 6xezE, 6xezF, 6zslA, 6zslB) of the ZBD domain to identify conformational states associated with the greatest ligand efficiency. This analysis identified three minor low energy minima “A,” “B,” and “C” for an aromatic pharmacophore in the binding region covered by the CHAPSO small-molecule (Fig 14B). The most favorable of these sites, “A” was in nearly an identical location (RMSD < 2.0 Å) to the previously identified Nsp13 Site 01 that was identified in our previous analysis as the most favorable site from the 6jytA structure. The cross-docking analysis of the six conformations of the ZBD identified specific conformational states of the helicase ZBD had higher ligand efficiencies using both pharmacophore docking and subsequent fragment screening. For the 6xez cryoEM structure, specifically the F chain conformation (LE = 0.37) had a much higher ligand efficiency for the same aromatic pharmacophore site than for the E chain (LE = 0.28), which is the CHAPSO ligand-bound induced conformation (Fig 14D). For the 6zsl high-resolution crystal structure, specifically the A chain conformation (LE = 0.35) had a higher ligand efficiency compared to the B chain (LE = 0.32). These conformational changes involve ZBD domain residues V45, L65, M68, F81, N86 and K94 as shown on (Fig 14A). Subsequent fragment screening for these sites using the FRAG1000 library also recapitulate the ranking of ligand efficiency from the pharmacophore mapping step. Within the first 1000 low MW fragment ligands screened (MW > 150), several fragment ligands were identified with favorable efficiency (0.40 < LE < 0.45) as shown on (Fig 14E). Thus, from our collective structural studies and analysis of the new replication transcription complex [61], we conclude that targeting Site 01 may be amenable to virtual screening and biophysical screening approaches.

**Fig 15:**
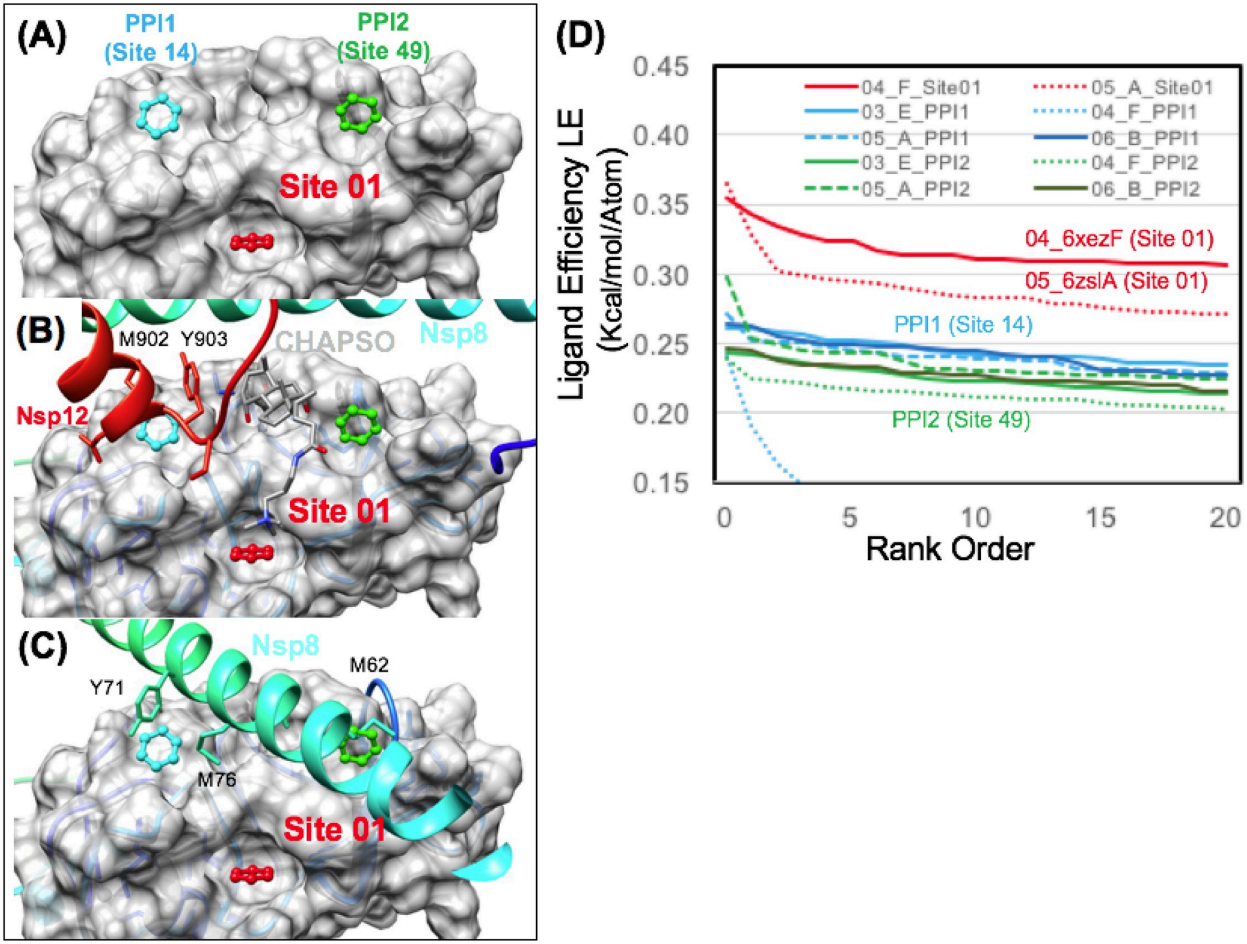
Nsp13 helicase ZBD allosteric Site 01 is more favorable than ZBD protein-protein interaction (PPI) sites. Pharmacophore Site 01 (red), Site 14 (cyan) and Site 49 (green) are shown on a surface diagram (A) of the ZBD. PPI1 (Site 14) overlaps with Nsp12-Nsp13 (B) and Nsp8-Nsp13 (C) PPI sites, where PPI2 (Site 49) overlaps with residue M62 of Nsp8 on the Nsp8-Nsp13 complex of the new replicase complex. Ligand efficiency from computational fragment screening (FRAG100) shown in (D) demonstrates that Site 01 (red) is more favorable than both of the PPI sites (cyan) and (green). Site 01 (red) was found to be most favorable for ligand binding in two new independently determined SARS-CoV-2 [04_6xezF] and [05_6zslA] Nsp13 experimental structures.

In comparison, targeting the exact PPI site directly may not be as thermodynamically favorable as illustrated in (Fig 15). Two aromatic pharmacophore sites identified in our study shown in (Fig 15A) retrospectively matched newly identified Nsp12 and Nsp8 binding sites on the Nsp13 surface from the new replication transcription complex. Ligand efficiency at Site 01 or at the PPI1 (Site 14) or the PPI2 (Site 49) from the newly released structures of the Nsp13 were assessed using fragment screening. These studies showed that two of the new experimental structures of the Nsp13 ZBD domain showed much higher ligand efficiencies from fragment screening at Site 01 [04_6xezF_Site_01] and [05_6zslZA_Site_01] compared to any of the new Nsp13 ZBD domain structures at the PPI1 (Site 14) or the PPI2 (Site 49). These observations support the independent identification of the Site 01 location on multiple experimental structures of Nsp13. Using cross-docking analysis nomenclature from (Fig 14D), identification of Site 01 was not merely an artifact of the [01_6jytA] structure, but was also identified as favorable in two other new experimental structures [04_6xezF] and [05_6zslA] in both pharmacophore mapping (Fig 14D) and fragment screening characterization (Fig 14E) and (Fig 15D).

### S2 spike protein

Our studies of the S2 segment of the Spike protein (res:711–1147) used the 3.2 Å CryoEM structure (6vxx.pdb) of the full-length SARS-CoV-2 Spike protein where the ectodomain was in the “closed” state [75]. Compared to larger conformational changes in the ectodomain, the trimeric S2 segment (res:711–1147) that we focus on exhibits less structural deviations (Cα RMSD < 0.5 Å) with other available pre-fusion structures such as the corresponding CryoEM structure of the ectodomain “open state” [75, 116]. As expected, there is greater structural deviation (Cα RMSD < 1.2 Å) over S2 (res:711–1147) in the furin cleaved structure of Spike (6zgg.pdb) [117] and (C_a_ RMSD < 1.0 Å) in other furin cleavage resistant engineered constructs (6zge.pdb) of Spike [117]. Although other recent computational studies similar to ours have focused on structural changes in the N-terminal ectodomain and identifying promising pockets for virtual screening [118], we aimed to focus on mapping the trimeric structure of the S2 segment. As explicitly modeled glycosylation sites were not included in our all-atom models of the trimeric S2 protein, we carefully analyzed our results for the possibility of artifacts due to this assumption by comparing to a published all-atom model including known explicit glycosylation’s [119]. On the basis of this structural comparison, very few of the identified benzene binding sites were in direct proximity to a glycosylation site and none of the TOP50 sites had significant atom clashes with any glycosylated site. Thus, we conclude that the approximation likely had minimal effect on the direct identification of a favorable site, but it is possible that the effects of being proximal to a glycosylation could have a more marked effect on the predicted ΔG_bind_ and the energetic ranking of sites.

**Fig 16:**
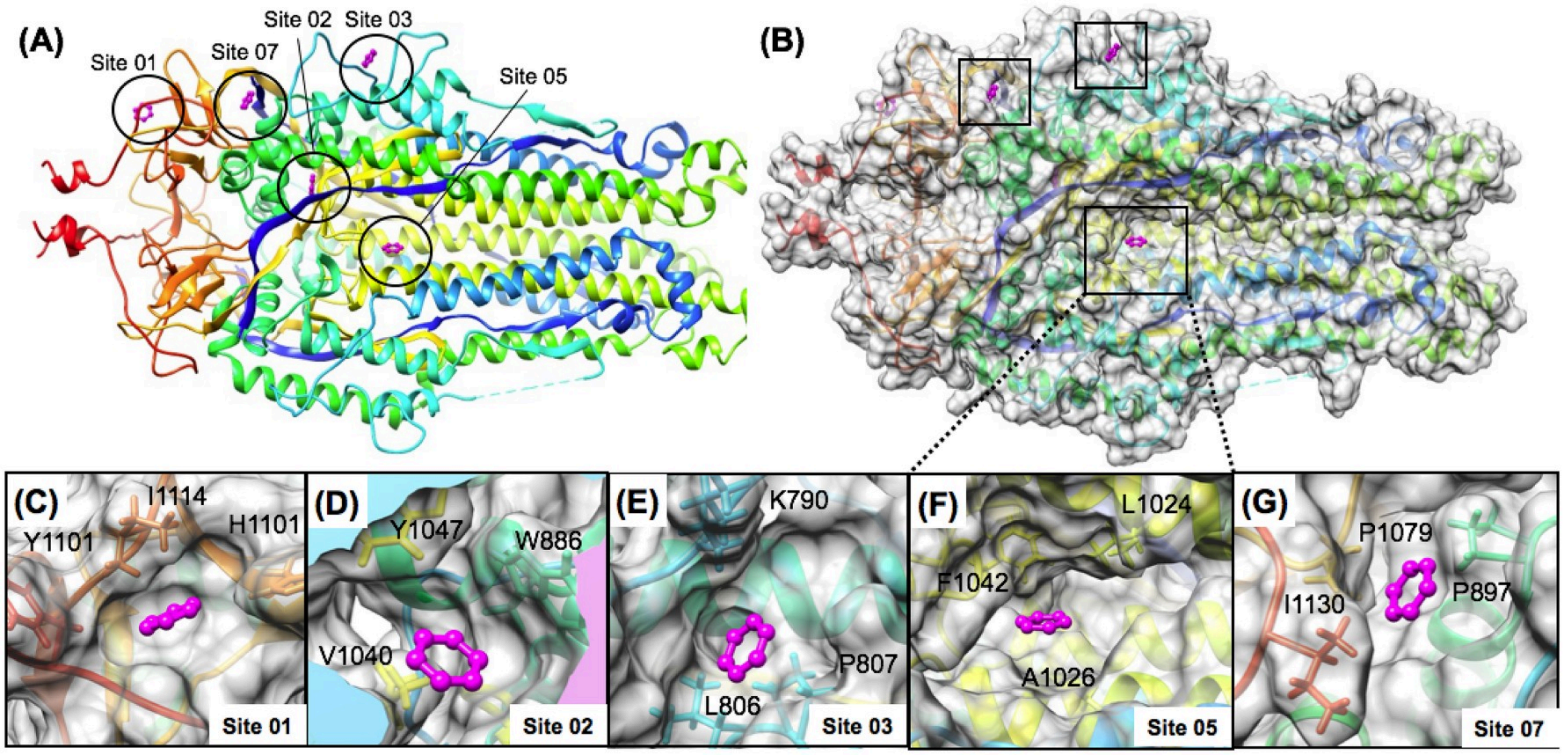
Favorable aromatic pharmacophore binding sites identified on the S2 spike protein. Favorable Sites 01, 02, 03, 05 and 07 are shown in magenta sites on a rainbow ribbon diagram in (A) and on a surface representation in (B). Important hydrophobic residues forming these sites are shown for Site 01 (C) Site 02 (D) Site 03 (E) Site 05 (F) and Site 07 (G).

**Fig 17:**
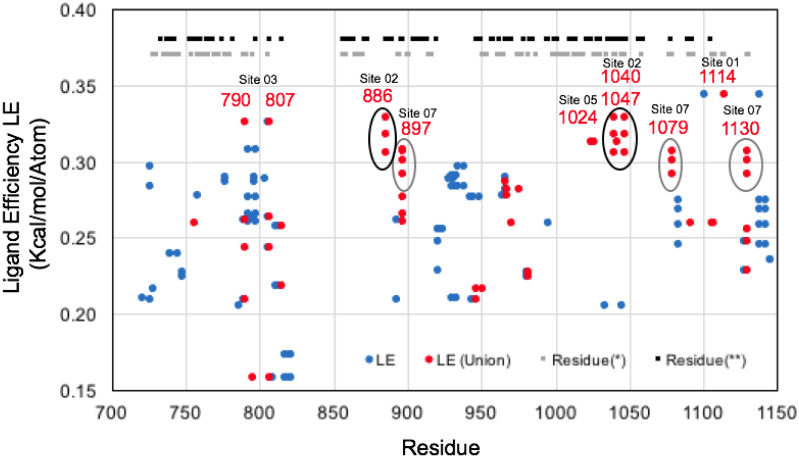
Ligand efficiencies from pharmacophore mapping of the S2 spike protein as a function of residue. All residues identified as having a high druggability and sequence-conservation by [120] are shown as small gray or black squares at the top of the figure. Black indicates a higher sequence conservation compared to gray between more distantly related coronaviruses (SARS, MERs, etc). Ligand efficiencies of the TOP50 sites (according to residues provided S1 Table in S1 File) are shown as either blue or red dots as a function of residue. Blue dots are pharmacophore sites (and corresponding residues) not identified in the analysis of Trigueiro-Louro et al., [120] whereas red dots indicate binding site residues identified as a Union of both datasets. The residues that define pharmacophore Sites 01, 02, 03, 05, and 07 exhibit high relative ligand efficiency to other sites on the S2 Spike protein and were also identified by Trigueiro-Louro et al., [120].

We present results for five favorable representative aromatic pharmacophore sites identified within the TOP50 S2 Spike segment (Fig 16). The S2 segment also has been recently analyzed in detail Trigueiro-Louro et al., to identify segments that are attractive to target with regards to sequence/structure conservation between beta coronavirus strains as well as predicted druggability by residue [120]. All five of the favorable sites reported (Fig 16) were also predicted within specific sequence regions identified by Trigueiro-Louro et al., as having favorable druggability properties [120]. The overlap of our results for S2 and the residues identified by Trigueiro-Louro et al., [120] are shown in (Fig 17).

Numerous binding sites within the TOP50 for the trimeric S2 spike protein were identified that were 3-fold symmetric, however there were minor differences in the predicted ΔG_bind_ for various sites. In the pharmacophore mapping step, the Site 01 (LE = 0.34) was identified as the most favorable site over the entire S2 trimeric structure (Fig 16). While there were several other favorable sites distributed along the S2 segment, the three dimensional architecture of this very C-terminal site in the CP.1 domain binding site did not provide obvious rational as to why strong ligand binding at that site would prevent membrane fusion conformational change. Site 01 did contain one residue I1114 (Fig 17) identified in [120].

The next most favorable binding site was Site 02 (LE = 0.33) which is an extremely buried site in the central cavity of the trimeric structure (Fig 16D). As shown in (Fig 17), Site 02 is composed of three residues (W886, V1040, Y1047) that were all identified in the analysis of Trigueiro-Louro et al., [120]. Of sites in the TOP50 that were robustly identified three times in 3-fold symmetric binding sites on the trimer, Site 02 was the most favorable. Similar to Site 02, Site 07 (Fig 16G) was also identified to be favorable three times in 3-fold symmetric binding sites on the structure of the trimer. Site 07 (Fig 17) is composed of three residues (P897, P1079, I1130) that were also all identified in [120]. In summary, in comparing the overlap of our results with Trigueiro-Louro et al., [120] by residue (Fig 17), Site 02 and Site 07 are the strongest matches by sequence, as both are composed of at least three important residues that overlap between the two datasets.

The next most favorable site that we report, Site 05 (LE = 0.31) was of interest for several reasons, but our comparison to the recent results of Trigueiro-Louro et al., [120], highlighted the importance to us of residues 885–891 and 1036–1048. These residues are colored magenta in (S5 Fig in S1 File), and are both flexible loops with conserved and druggable residues [120]. Inhibitors that may bind in this region are hypothesized to interfere with the S2 fusion conformational change machinery.

Using a structural alignment (structure-sequence) superposition of our S2 spike protein with influenza (5t6n.pdb and 5t6s.pdb), we show where the influenza fusion inhibitor Arbidol most likely binds to the S2 spike, within close proximity to Site 05. Using the UCSF Chimera Matchmaker structural alignment algorithm [81, 82], a superior structural alignment was achieved using our S2 trimeric (6vxx.pdb) as a query matching the structure of (5t6s.pdb) compared to (5t6n.pdb) [121]. This resulted in a reasonable structural alignment of 20 residues (Cα RMSD < 0.5 A) over a stretch of 25 residues with very good matches to secondary structural elements. This superposition of where Arbidol binds to influenza projected onto the structure of the S2 segment allows only a rough approximation of where it most likely binds on the S2 segment, as shown in (S5 Fig in S1 File).

The results were also compared with the same protocol using reference “knowledge-based” pharmacophores where a total of 12 reference aromatic pharmacophore positions for Arbidol binding were derived from the structural alignment with (5t6s.pdb) [121]. When these were ranked by (ΔG_bind_) the most favorable of the knowledge-based sites binding sites (LE = 0.27) was still not as favorable as the previously identified Site 05 (LE = 0.31). Therefore, within close proximity to some of the knowledge-based references, Site 05 is more favorable. The closest of the reference pharmacophores was within (5.7 Å) of Site 05, which also supports this as a likely location for Arbidol binding.

Another independent report has predicted that Arbidol binds to the S2 segment within proximity to Site 05 [122]. Using molecular docking techniques, Vankadari predicted that Arbidol bound to the S2 segment in proximity to residues 776, 780, 1017, 1019, 1021, 1023, 1024, 1027 (colored in cyan) shown in (S5 Fig in S1 File). In this work, Vankadari proposed that Arbidol may act as a direct trimerization inhibitor [122]. Other laboratories have also proposed targeting inhibition of trimerization as a strategy to target Spike [123]. It seems that effective small-molecule binding to Site 05 (on the trimer) or the nearby site identified by Vankadari [122] would most likely exhibit a mechanism of action similar to Arbidol, targeting the hemagglutinin fusion conformational change machinery. Arbidol specifically was shown to stabilize the semi-stable prefusion conformational state preventing conformational changes associated with membrane fusion [121].

Interestingly, recent crystal structures of two other class I fusion protein inhibitors show that binding sites at several locations on a trimeric prefusion conformation may lead to inhibition. In the structure of the respiratory syncytial virus (RSV) F glycoprotein, several small-molecule inhibitors were found to bind to a three-fold symmetric pocket within the central cavity [124]. The Ebola Virus glycoprotein was found to bind the fusion inhibitor toremifene in a pocket between the GP1 attachment and GP2 fusion subunits [125]. Thus, there is structural evidence for small-molecule fusion inhibitors to bind and inhibit fusion at more than one site. The buried Site 02 reported here is similar to the RSV inhibitors binding site in that the sites are deeply buried within the central cavity. Site 05 is similar to the influenza HA Arbidol binding site by our structural alignment and as predicted by docking by Vankadari [122]. Another similarity, with regards to quaternary structure is that Site 05 also is on the monomer-monomer interface of the trimer, similar to how Arbidol binds to influenza HA on the interface of monomer 1 and monomer 2 [121].

Of the five favorable sites that we focus on here, Site 05 site seems to be reasonably promising with regards to being proximal to where Arbidol most likely binds, as independently identified and predicted by other laboratories and computational techniques [121]. For these reasons, we also benchmarked Site 05 on the S2 Spike protein with fragment screening for the entire FRAG3700 library. Interestingly, recapitulating the previous results from screening the smaller version of this library, screening the entire library demonstrated that this site had much lower ligand efficiencies (LE_MAX_ = 0.30) compared to the most favorable sites on Mprot (LE_MAX_ = 0.50), similar to our fragment screening data for the Nsp13 helicase. Thus, the most favorable site identified within proximity of where Arbidol likely binds shown in (Fig 16F) and (S5 Fig in S1 File) still has a relatively low ligand efficiency compared to the most favorable sites on Nsp5 Mpro, Nsp12 RdRp, or Nsp16 2’-O MT.

Arbidol has been shown by independent laboratories to act as a weak (~3.5 μM) inhibitor of SARS-CoV-2 virus *in vitro* replication assays [126, 127]. Arbidol has also been reported to have superior efficacy in comparison to lopinavir/ritonavir in small clinical trials in China [128]. Unfortunately, we found the majority of the sites on the Spike protein to have lower ligand efficiencies than other SARS-CoV-2 targets studied here. Despite that fact, it still appears encouraging that improved derivatives of Arbidol will be developed in the future that have improved potency for the SARS-CoV-2 Spike protein.

## Conclusions

Our approach of pharmacophore mapping and fragment screening allows a comparison of the most favorable sites among the targets studied in detail. Our calculated ligand efficiencies from each iterative step consistently suggest that Nsp5 Mpro, Nsp12 RdRp, and Nsp16 2’-O MT have more favorable binding sites with greater ligand efficiency compared to Nsp13 helicase and the S2 Spike protein. Pharmacophore mapping results for Nsp5 Mpro correctly identified three of the most favorable peptide side chain substrate recognition sites, which confirmed the accuracy of our methodology. Independent experimental fragment screening structural data for Nsp5 Mpro was able to experimentally corroborate aromatic pharmacophore locations for three additional predicted “minor” fragment ligand binding sites outside of the protease active site.

In reviewing the pharmacophore mapping results for the target proteins, it is apparent that many of the most favorable sites happen to be areas that correspond to protein-protein interactions (PPIs) of different constituents of the replicase complex. Nsp12 was found to have the most favorable site (LE = 0.48) which corresponded to the most important Nsp8 binding interaction. The most favorable PPI site identified for Nsp13 (LE = 0.22) has a far lower LE when compared to Nsp12. The most favorable PPIs in the Nsp16:Nsp10 complex were Site 03 (LE = 0.31) and Site 06 (LE = 0.27), in comparison. Therefore, some of the most favorable sites that undergo protein-protein interactions could be targeted to inhibit assembly of the active replicase complex. Our analysis points to the Nsp12 RdRp Site 01 (LE = 0.48), as the single PPI site with the highest ligand efficiency (Fig 9).

Our results have shown that the Nsp13 helicase does not have the most thermodynamically favorable sites to target in comparison to several of the other examined targets. However, while these data suggest that targeting the function of the helicase itself may be difficult, there is promise in targeting the helicase to prevent the formation of the replication complex. Fragment screening at numerous sites on Nsp13 helicase identified several favorable sites on the N-terminal ZBD. Site 01 (LE = 0.31) was found to be the single most favorable site on the entire SARS-CoV helicase structure (6jyt.pdb). Fragment screening data (Fig 14E) demonstrates that this favorable Site 01 also exists on two new independent structures (6xez.pdb) (6xsl.pdb) of the SARS-CoV-2 Nsp13 helicase. The small-molecule CHAPSO was recently found to bind in the vicinity of this site in a new CryoEM structure (6xez.pdb), experimentally confirming ligand binding at our previously identified Site 01 [61]. We propose that ligand binding targeting this favorable N-terminal site of the Nsp13 helicase may induce minor conformational changes in the ZBD that inhibit function or prevent the assembly of the replication complex by blocking PPIs of binding domains.

This comparison of sites between SARS-CoV-2 drug target proteins highlights that there are numerous favorable sites to target with small-molecules via virtual screening or biophysical screening approaches. However, due to differences in physiochemical properties and molecular architecture, some small-molecule binding sites are more favorable than others. It is our hope that insights from this work will be helpful to advance the development of preclinical candidates for each of these targets.

## Supporting information

S1 File (DOCX)

S2 File (ZIP)
