# Supplementary material for: Mapping major SARS-CoV-2 drug targets and assessment of druggability using computational fragment screening: Identification of an allosteric small-molecule binding site on the Nsp13 helicase
Source: PLoS One. 2021 Feb 17;16(2):e0246181. doi: 10.1371/journal.pone.0246181 (PMC7888625; doi:10.1371/journal.pone.0246181)
Supplement: S1 File — (DOCX) [file pone.0246181.s001.docx]

**S1 File**

Supporting information is provided for the structural map of aromatic pharmacophore sites and calculated ligand efficiencies. Data for these are provided as available supplementary information in the form of detailed data table (S1 Table) at the end of supporting information and a corresponding .zip file of associated .pdb files (S2 File). Every entry in the table (S1 Table) represents a .pdb file in supplemental (S2 File) for aromatic pharmacophore sites on a specific target protein.

**S1 Fig.** **Aromatic heterocycle replacement library.** Library is composed of representative cmps that contain specific substructures shown for Group 2, 3, 4 and 6.

**
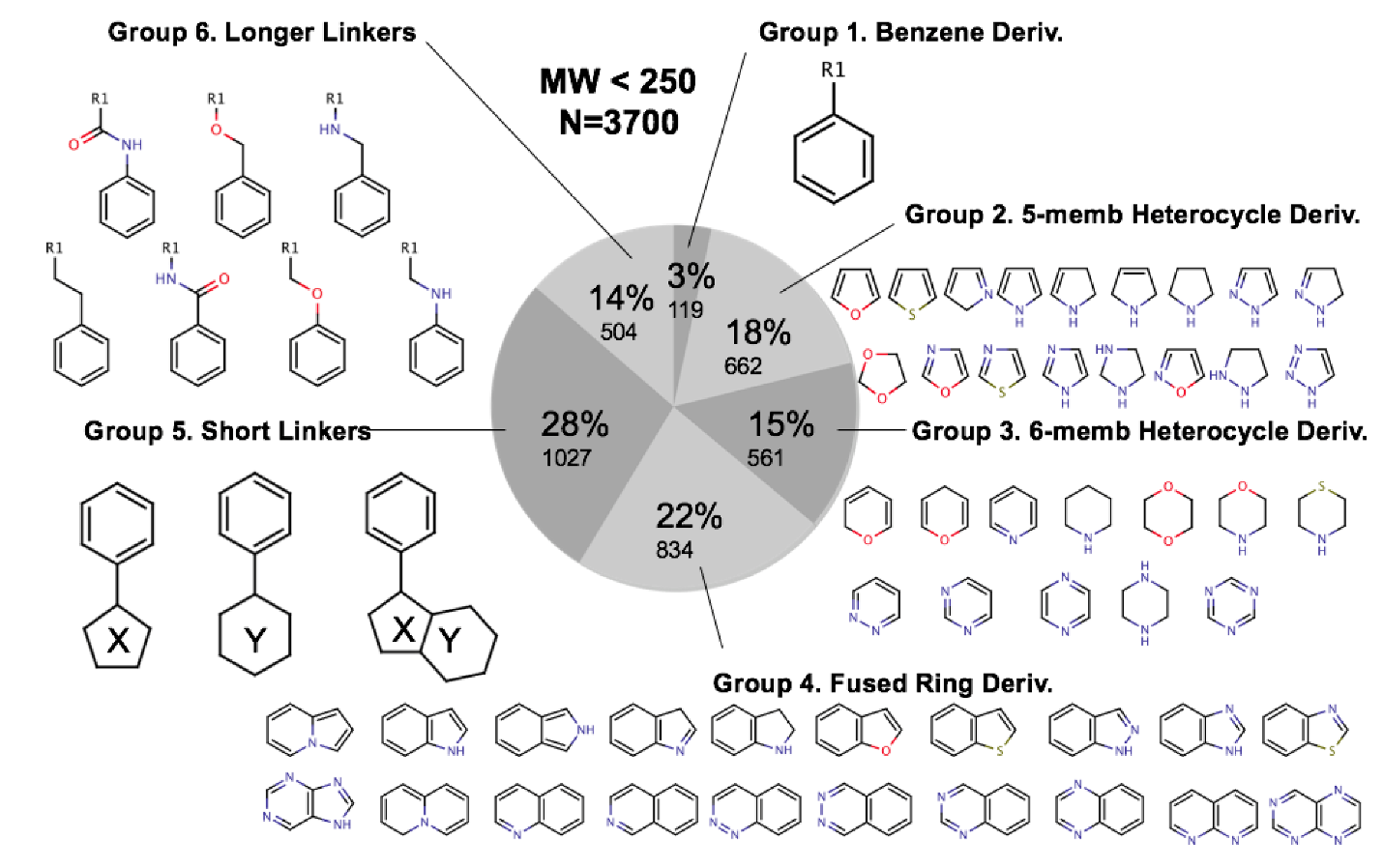
**

**S2 Fig.** **Library composition and distribution of physiochemical properties.** (A) Molecular Weight (B) Heavy Atoms (C) cLogP (D) Rotatable Bonds.


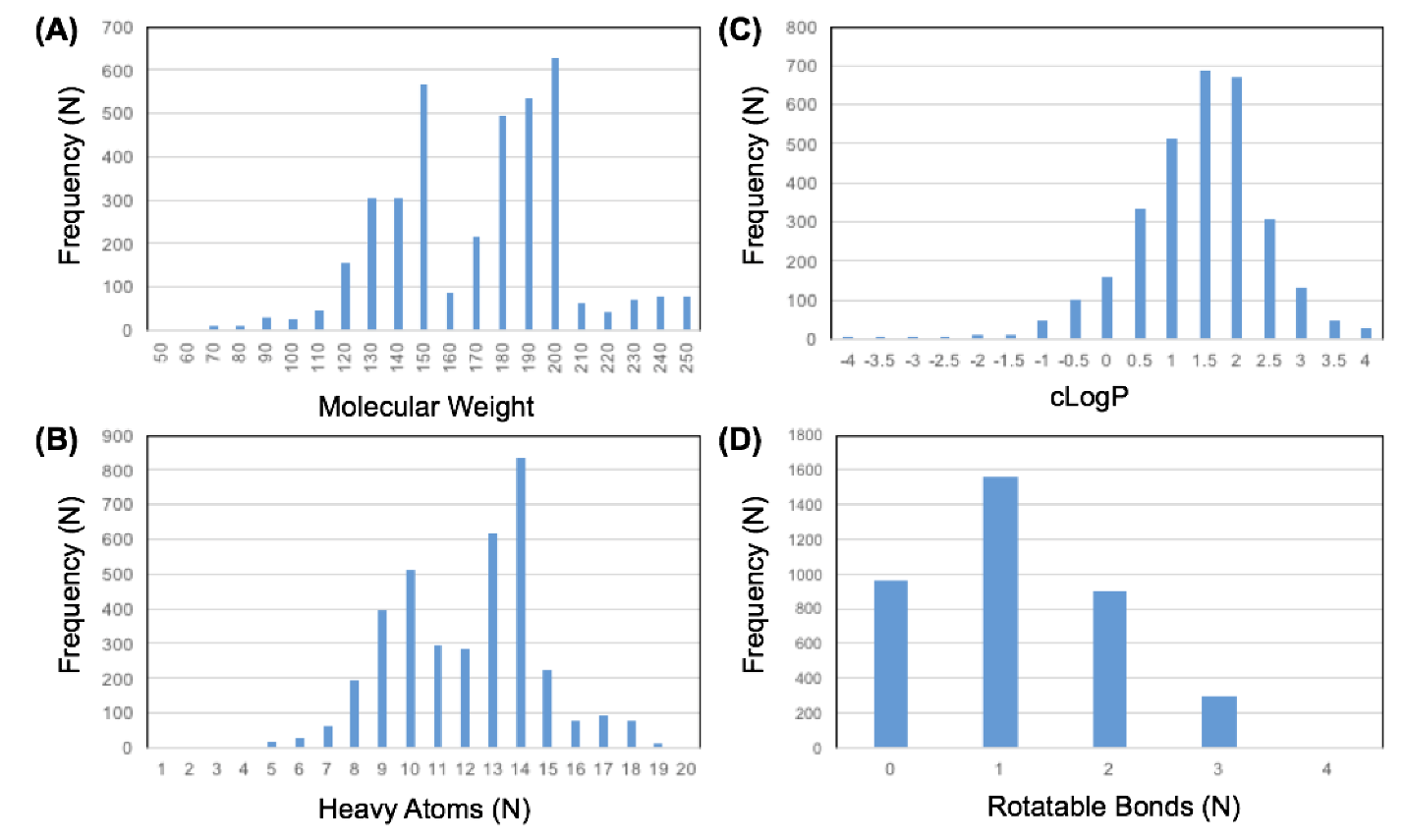


**S3 Fig.** Ligand efficiency comparisons from computational fragment screening. Ligand efficiency comparing numerous sites on different target proteins using the FRAG100 library as a benchmark: (A) Nsp5 Mpro (B) Nsp16 2’-O MT (C) Nsp12 RdRp (D) Nsp13 Helicase (E) S2 Spike. Ligand efficiency data is also shown as a function of fragment molecular weight and calculated LogP for rigorousness to assess library sampling of physiochemical property space.


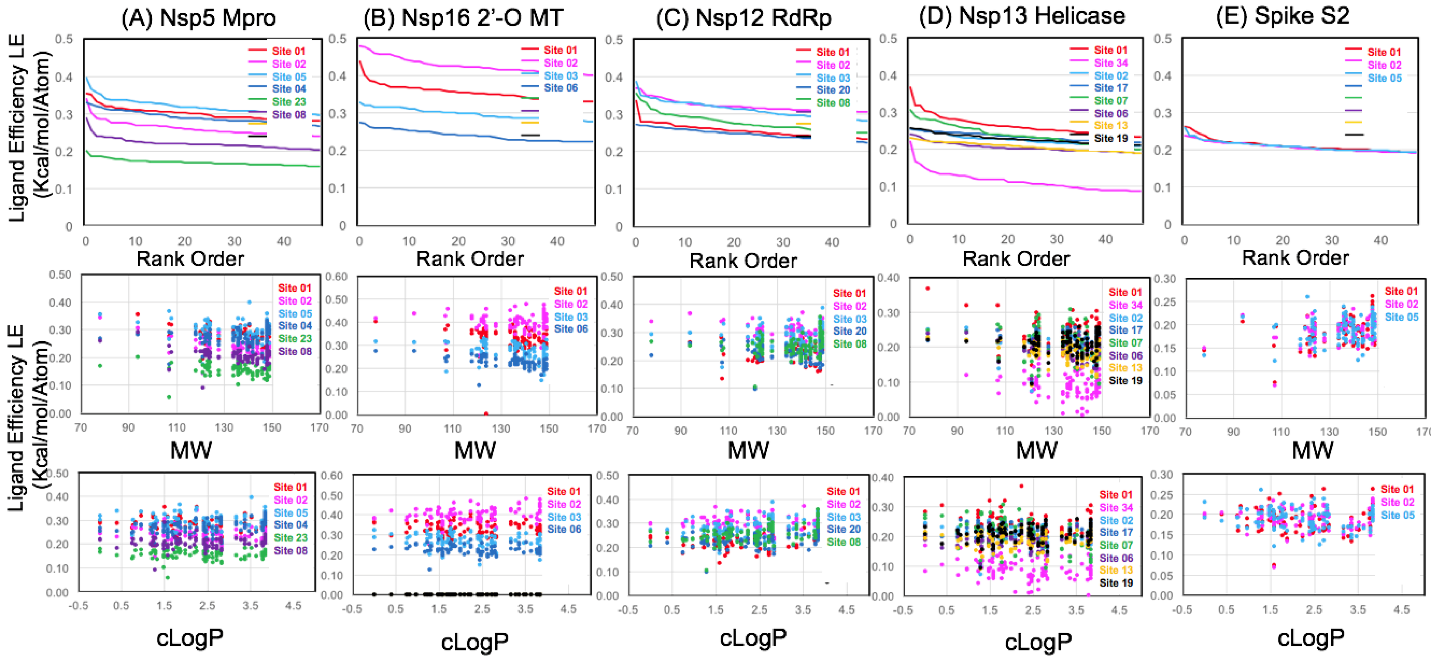


**S4 Fig.** **Example positive controls to assess fragment docking geometry.** Docking into the correctly identified Nsp5 Mpro Site 01 was able to recapitulate binding geometries of fragments with experimentally determined structures. Retrospectively, these are successful structural predictions from docking where 80% satisfy the strict criteria (RMSD < 2.0 Å) and 100% satisfy (RMSD < 2.5 Å).


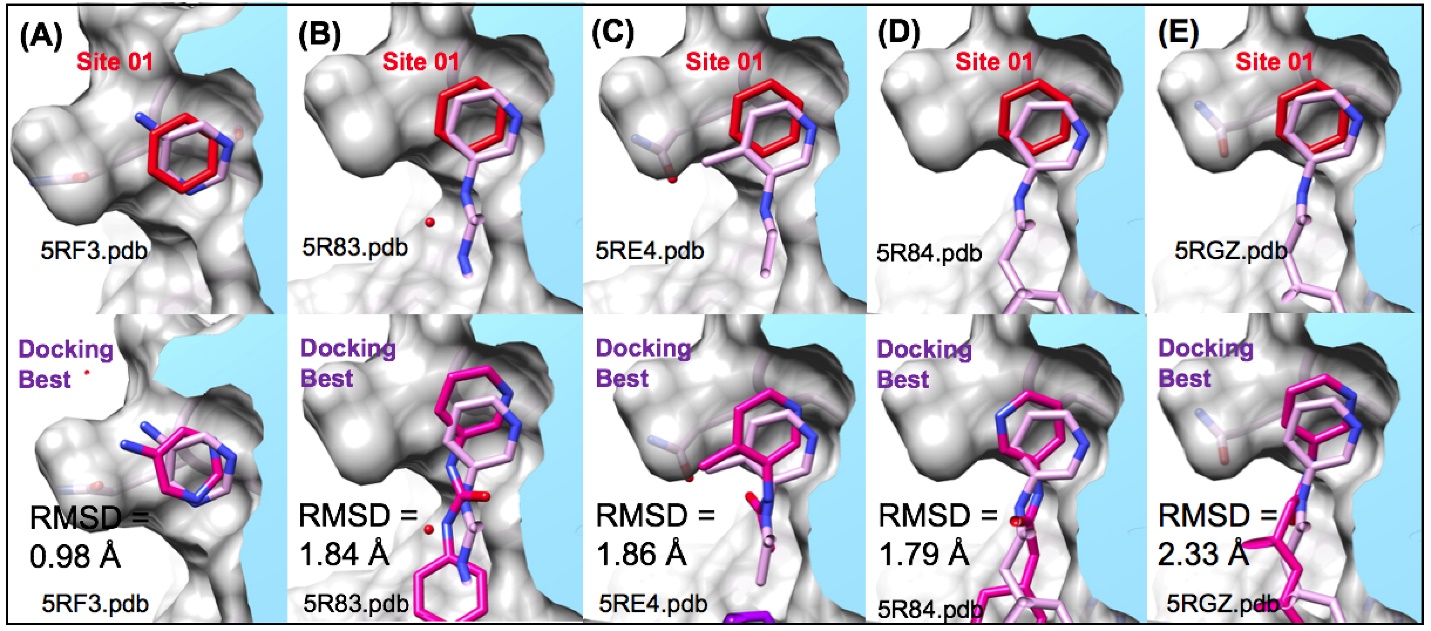


**S5 Fig.** **Favorable Site 05 on the S2 Spike protein is proximal to a reported Arbidol binding site.** Site 05 is shown in red on both a ribbon diagram of the trimer shown with gray coloring (A) and shown with a transparent gray surface in (B). Residues 885-891 and 1036-1048 (colored in magenta) are both flexible loops identified in the analysis of Trigueiro-Louro et el. [120] as having both conserved and druggable residues. The structural conformations of Arbidol shown are derived from a sequence-structural alignment of the SARS-CoV-2 and influenza virus structures where the residues matched are highlighted and shown in (B). Vankadari [122] predicted that Arbidol bound to the S2 segment in proximity to residues 776, 780, 1017, 1019, 1021, 1023, 1024, 1027 (colored in cyan). A zoom in view of the Site 05 pharmacophore site is shown in more detail on ribbon diagram (C) and on transparent gray surface (D). Site 05 is close in proximity of these residues colored in cyan and magenta respectively and where Arbidol may be reasonably assumed to bind.


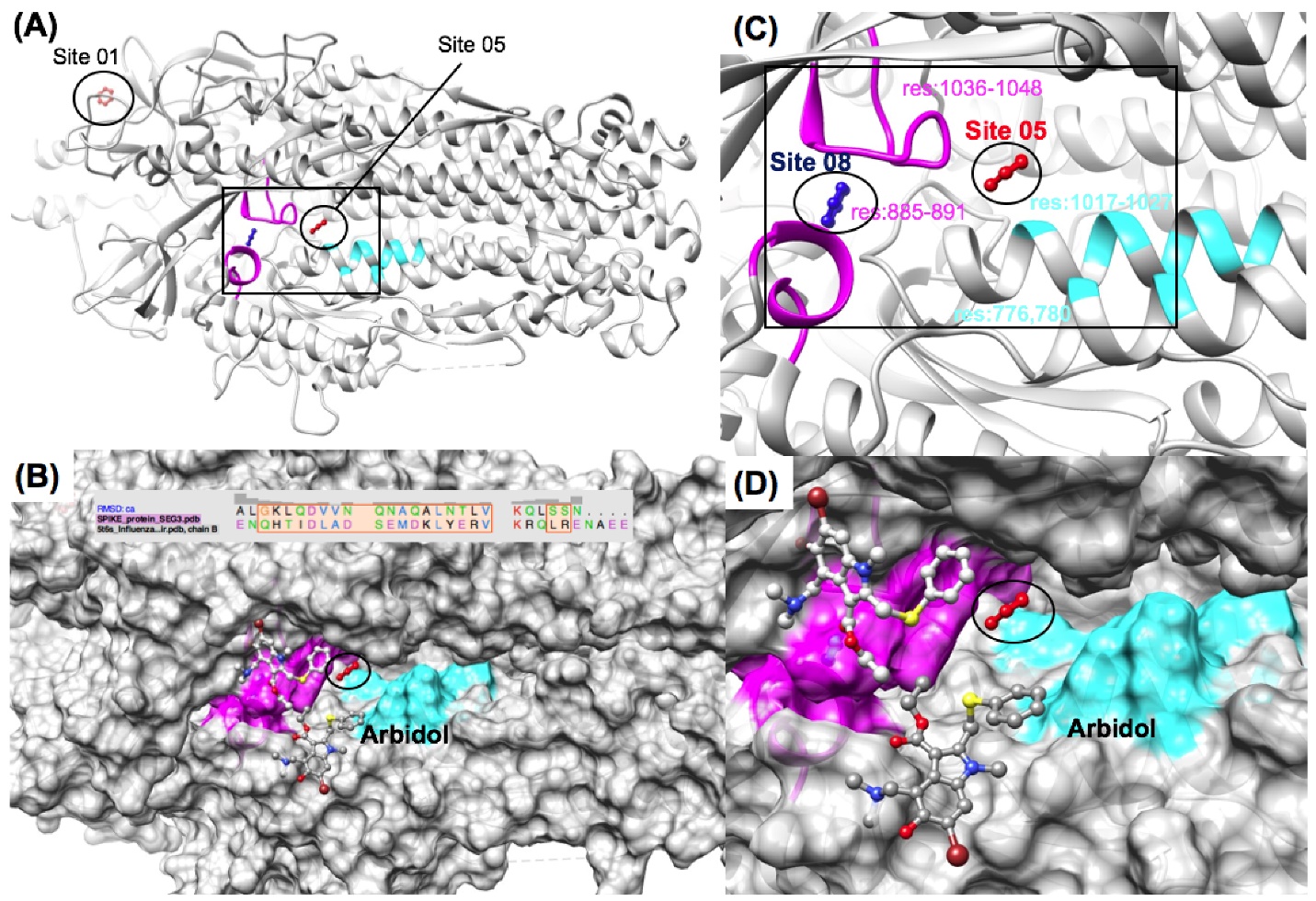


**S1 Table.** **Data for the structural map of aromatic pharmacophore sites and calculated ligand efficiencies on seven SARS-CoV-2 drug target proteins.** Either the TOP25 or TOP50 sites are reported for Nsp4, Nsp5, Nsp12, Nsp13, Nsp15, Nsp16, and the S2 Spike protein. Each entry in this table for a specific site and target protein correspond exactly to provided Supplementary PDB files of the aromatic pharmacophore sites and target protein structure analyzed. For each site in the table, the first identifier is the Site number (N) which is ranked by ΔG_bind_. Next, properties that are provided are ligand efficiency (LE) and ΔG_bind_ (Kcal/mol). For each site, the next identifier is denoted BEN, which is the number (N) of the ranked list of benzene binding sites ranked by the number of hydrophobic contacts. The number of hydrophobic contacts are also reported for each site using the criteria X. This table does not provide an exhaustive map of sites that overlap with known protein-protein interaction (PPI) sites, but for target sites on (Nsp12, Nsp13, and Nsp16) specifically discussed and analyzed in the text as PPI sites are denoted with a ** symbol. For each site reported in the table, three hydrophobic residues are reported on the target protein that form numerous hydrophobic contacts with the pharmacophore and are essential contacts forming the 3D architecture of the binding site. For the larger proteins (Nsp12, Nsp13, and S2 Spike) entries are also added to the table for “knowledge-based” aromatic pharmacophore sites that are derived from structural alignments with reference .pdb files. These “knowledge-based” entries in the table are denoted REF_ followed by the corresponding pdb ID, such that each entry in this table correspond exactly to provided Supplementary PDB files.

| Nsp4 (Papin-like) | Site (N) | LE | dG (Kcal/mol) | BEN | Conts (N) | N/A | Res 1 | Res 2 | Res 3 |
| --- | --- | --- | --- | --- | --- | --- | --- | --- | --- |
|  | 1 | 0.35 | -2.07 | BEN_05 | 72 |  | T74 | D76 | Y154 |
|  | 2 | 0.31 | -1.87 | BEN_02 | 76 |  | T257 | Y305 | K306 |
|  | 3 | 0.31 | -1.86 | BEN_14 | 55 |  | V187 | I222 | T225 |
|  | 4 | 0.30 | -1.78 | BEN_01 | 76 |  | S85 | T158 | V159 |
|  | 5 | 0.28 | -1.68 | BEN_04 | 72 |  | R65 | T75 | P77 |
|  | 6 | 0.27 | -1.63 | BEN_03 | 74 |  | P247 | P248 | Y264 |
|  | 7 | 0.26 | -1.53 | BEN_23 | 51 |  | T311 | T312 | K315 |
|  | 8 | 0.25 | -1.51 | BEN_08 | 62 |  | W106 | H272 | L289 |
|  | 9 | 0.25 | -1.49 | BEN_13 | 55 |  | Y251 | K254 | Y305 |
|  | 10 | 0.24 | -1.45 | BEN_22 | 51 |  | L178 | D179 | V202 |
|  | 11 | 0.24 | -1.44 | BEN_09 | 58 |  | F31 | V57 | L64 |
|  | 12 | 0.23 | -1.36 | BEN_07 | 66 |  | V2 | R3 | M23 |
|  | 13 | 0.23 | -1.36 | BEN_24 | 50 |  | K157 | E161 | L162 |
|  | 14 | 0.23 | -1.36 | BEN_06 | 68 |  | E238 | P240 | N308 |
|  | 15 | 0.22 | -1.33 | BEN_15 | 54 |  | V235 | K315 | P316 |
|  | 16 | 0.22 | -1.33 | BEN_16 | 53 |  | P248 | T265 | P299 |
|  | 17 | 0.22 | -1.29 | BEN_11 | 56 |  | K6 | F8 | T18 |
|  | 18 | 0.22 | -1.29 | BEN_12 | 56 |  | K217 | K218 | I314 |
|  | 19 | 0.21 | -1.27 | BEN_25 | 50 |  | Q174 | V202 | E203 |
|  | 20 | 0.20 | -1.21 | BEN_21 | 51 |  | R65 | V66 | F69 |
|  | 21 | 0.19 | -1.15 | BEN_17 | 53 |  | E124 | K126 | Y136 |
|  | 22 | 0.19 | -1.15 | BEN_18 | 53 |  | K254 | H255 | E295 |
|  | 23 | 0.17 | -1.04 | BEN_19 | 52 |  | Q19 | Q30 | F31 |
|  | 24 | 0.17 | -1.01 | BEN_20 | 52 |  | T210 | V220 | Q221 |
|  | 25 | 0.11 | -0.65 | BEN_10 | 57 |  | V188 | Y233 | P316 |
| Nsp5 (Mpro) | Site (N) | LE | dG (Kcal/mol) | BEN | Conts (N) | N/A | Res 1 | Res 2 | Res 3 |
|  | 1 | 0.36 | -2.16 | BEN_03 | 74 |  | E166 | F140 | N142 |
|  | 2 | 0.34 | -2.04 | BEN_05 | 73 |  | V35 | K88 | K90 |
|  | 3 | 0.33 | -1.96 | BEN_01 | 81 |  | F3 | L282 | F291 |
|  | 4 | 0.32 | -1.90 | BEN_09 | 65 |  | M165 | P168 | Q189 |
|  | 5 | 0.31 | -1.89 | BEN_08 | 69 |  | H41 | M49 | Q189 |
|  | 6 | 0.30 | -1.82 | BEN_18 | 54 |  | L272 | M276 | L287 |
|  | 7 | 0.30 | -1.80 | BEN_06 | 72 |  | W218 | E270 | L271 |
|  | 8 | 0.26 | -1.59 | BEN_11 | 62 |  | T189 | M235 | P241 |
|  | 9 | 0.25 | -1.52 | BEN_16 | 56 |  | V247 | A260 | V261 |
|  | 10 | 0.25 | -1.49 | BEN_17 | 55 |  | D48 | L50 | N51 |
|  | 11 | 0.24 | -1.47 | BEN_02 | 75 |  | F223 | A266 | K269 |
|  | 12 | 0.24 | -1.46 | BEN_14 | 60 |  | N180 | F181 | V186 |
|  | 13 | 0.24 | -1.42 | BEN_19 | 54 |  | F3 | I213 | C300 |
|  | 14 | 0.23 | -1.39 | BEN_13 | 61 |  | Q47 | L50 | N51 |
|  | 15 | 0.22 | -1.31 | BEN_25 | 50 |  | V233 | K236 | Q273 |
|  | 16 | 0.21 | -1.23 | BEN_21 | 52 |  | M276 | L286 | L287 |
|  | 17 | 0.20 | -1.23 | BEN_07 | 69 |  | F223 | K269 | E270 |
|  | 18 | 0.20 | -1.19 | BEN_22 | 50 |  | E47 | M49 | L50 |
|  | 19 | 0.18 | -1.09 | BEN_20 | 52 |  | E55 | M82 | N84 |
|  | 20 | 0.18 | -1.07 | BEN_24 | 50 |  | L220 | R222 | I259 |
|  | 21 | 0.18 | -1.06 | BEN_04 | 73 |  | P52 | Y54 | R188 |
|  | 22 | 0.18 | -1.06 | BEN_23 | 50 |  | T226 | L227 | L262 |
|  | 23 | 0.17 | -1.01 | BEN_10 | 63 |  | R105 | P108 | F134 |
|  | 24 | 0.16 | -0.98 | BEN_12 | 62 |  | P9 | L12 | F305 |
|  | 25 | 0.08 | -0.47 | BEN_15 | 57 |  | K137 | L286 | E290 |
| Nsp12 (RdRp) | Site (N) | LE | dG (Kcal/mol) | BEN | Conts (N) | PPI (**) | Res 1 | Res 2 | Res 3 |
|  | 1 | 0.48 | -2.90 | BEN 02 | 79 | ** | F368 | L371 | W509 |
|  | 2 | 0.35 | -2.12 | BEN 03 | 75 |  | R349 | P461 | P677 |
|  | 3 | 0.34 | -2.02 | BEN 09 | 67 |  | L172 | L460 | P461 |
|  | 4 | 0.33 | -1.95 | BEN 22 | 59 |  | V410 | L544 | Y446 |
|  | 5 | 0.32 | -1.89 | BEN 08 | 69 |  | T604 | T607 | R750 |
|  | 6 | 0.31 | -1.88 | BEN 21 | 59 |  | F766 | I779 | T801 |
|  | 7 | 0.30 | -1.79 | BEN 07 | 69 |  | K574 | I488 | I494 |
|  | 8 | 0.30 | -1.79 | BEN 34 | 54 | ** | F396 | V398 | M666 |
|  | 9 | 0.28 | -1.69 | BEN 01 | 83 |  | K143 | V147 | F157 |
|  | 10 | 0.28 | -1.67 | BEN 05 | 72 |  | L389 | K391 | F396 |
|  | 11 | 0.28 | -1.67 | BEN 12 | 66 | ** | F429 | E431 | E436 |
|  | 12 | 0.27 | -1.61 | BEN 04 | 75 |  | W268 | L282 | F321 |
|  | 13 | 0.27 | -1.61 | BEN 23 | 59 |  | K807 | M818 | P873 |
|  | 14 | 0.26 | -1.59 | BEN 31 | 55 |  | F334 | N336 | H362 |
|  | 15 | 0.26 | -1.58 | BEN 29 | 55 |  | V231 | P232 | Y289 |
|  | 16 | 0.26 | -1.57 | BEN 50 | 50 |  | L187 | V190 | R285 |
|  | 17 | 0.26 | -1.57 | BEN 36 | 53 |  | P94 | A195 | D194 |
|  | 18 | 0.25 | -1.52 | BEN 11 | 66 | ** | F429 | L437 | F440 |
|  | 19 | 0.25 | -1.49 | BEN 06 | 71 |  | R305 | L470 | R735 |
|  | 20 | 0.25 | -1.48 | BEN 15 | 63 | ** | L271 | Y273 | L329 |
|  | 21 | 0.25 | -1.48 | BEN 10 | 66 |  | V299 | V353 | H355 |
|  | 22 | 0.25 | -1.48 | BEN 32 | 55 |  | K160 | W162 | Y163 |
|  | 23 | 0.24 | -1.47 | BEN 44 | 51 |  | K574 | K577 | A581 |
|  | 24 | 0.24 | -1.45 | BEN 27 | 57 |  | K511 | R513 | L514 |
|  | 25 | 0.24 | -1.44 | BEN 35 | 53 |  | F584 | I864 | S871 |
|  | 26 | 0.23 | -1.41 | BEN 24 | 58 |  | P322 | W286 | K267 |
|  | 27 | 0.23 | -1.40 | BEN 19 | 60 |  | M855 | L891 | E894 |
|  | 28 | 0.22 | -1.34 | BEN 16 | 63 | ** | P412 | F440 | F442 |
|  | 29 | 0.22 | -1.33 | BEN 20 | 59 |  | K849 | L854 | R858 |
|  | 30 | 0.22 | -1.32 | BEN 49 | 50 |  | V860 | Y915 | F920 |
|  | 31 | 0.22 | -1.31 | BEN 28 | 56 | ** | K391 | L388 | V405 |
|  | 32 | 0.22 | -1.31 | BEN 47 | 50 |  | I847 | V848 | T850 |
|  | 33 | 0.21 | -1.28 | BEN 30 | 55 |  | T736 | V742 | K478 |
|  | 34 | 0.20 | -1.22 | BEN 14 | 66 |  | I37 | V42 | F48 |
|  | 35 | 0.20 | -1.21 | BEN 39 | 52 |  | Q573 | L576 | K577 |
|  | 36 | 0.20 | -1.20 | BEN 40 | 52 |  | H599 | V827 | P927 |
|  | 37 | 0.20 | -1.17 | BEN 33 | 54 |  | F283 | Y294 | L280 |
|  | 38 | 0.19 | -1.14 | BEN 17 | 61 |  | P260 | M794 | K798 |
|  | 39 | 0.19 | -1.12 | BEN 42 | 52 |  | K411 | Y546 | I847 |
|  | 40 | 0.19 | -1.11 | BEN 37 | 53 |  | F35 | I37 | F48 |
|  | 41 | 0.18 | -1.10 | BEN 18 | 60 |  | K641 | T643 | D484 |
|  | 42 | 0.18 | -1.08 | BEN 13 | 66 |  | C487 | F528 | A529 |
|  | 43 | 0.18 | -1.07 | BEN 41 | 52 |  | T591 | S592 | Y595 |
|  | 44 | 0.17 | -1.04 | BEN 43 | 51 |  | F793 | E796 | A797 |
|  | 45 | 0.17 | -1.02 | BEN 38 | 52 | ** | F415 | F440 | F843 |
|  | 46 | 0.17 | -1.01 | BEN 25 | 57 |  | K369 | Y530 | R533 |
|  | 47 | 0.16 | -0.97 | BEN 48 | 50 |  | I333 | N360 | S363 |
|  | 48 | 0.10 | -0.61 | BEN 45 | 51 |  | R640 | K641 | T643 |
|  | 49 | 0.08 | -0.50 | BEN 26 | 57 |  | K272 | E274 | E277 |
|  | 50 | 0.08 | -0.49 | BEN 46 | 51 |  | Y32 | F48 | F49 |
| REF_7bv2 | 1 | 0.32 | -1.91 | REF_04 | N/A |  | D623 | T680 | T687 |
| REF_7bv2 | 2 | 0.22 | -1.29 | REF_03 | N/A |  | R555 | V557 | S682 |
| REF_3cwj | 3 | 0.21 | -1.24 | REF_06 | N/A |  | K593 | W598 | C813 |
| REF_3cwj | 4 | 0.20 | -1.20 | REF_05 | N/A |  | A688 | N691 | S759 |
| REF_5f3z | 5 | 0.18 | -1.09 | REF_01 | N/A |  | P832 | I837 | R836 |
| REF_5f3z | 6 | 0.15 | -0.90 | REF_02 | N/A |  | K593 | W598 | C813 |
| Nsp13 (Helicase) | Site (N) | LE | dG (Kcal/mol) | BEN | Conts (N) | PPI (**) | Res 1 | Res 2 | Res 3 |
|  | 1 | 0.31 | -1.88 | BEN 01 | 82 |  | F81 | A85 | F90 |
|  | 2 | 0.25 | -1.50 | BEN 07 | 63 |  | I20 | H39 | W114 |
|  | 3 | 0.25 | -1.47 | BEN 12 | 59 |  | A18 | K40 | L41 |
|  | 4 | 0.24 | -1.44 | BEN 05 | 67 |  | I249 | Y253 | P254 |
|  | 5 | 0.24 | -1.44 | BEN 46 | 45 |  | R442 | H464 | K462 |
|  | 6 | 0.24 | -1.44 | BEN 15 | 56 |  | E128 | R129 | L132 |
|  | 7 | 0.24 | -1.43 | BEN 11 | 60 |  | K288 | A313 | A316 |
|  | 8 | 0.23 | -1.40 | BEN 08 | 62 |  | R161 | Y211 | R212 |
|  | 9 | 0.23 | -1.38 | BEN 34 | 49 |  | K329 | S331 | F346 |
|  | 10 | 0.23 | -1.38 | BEN 48 | 44 |  | K430 | P434 | N459 |
|  | 11 | 0.23 | -1.37 | BEN 16 | 55 |  | V558 | L581 | K584 |
|  | 12 | 0.23 | -1.36 | BEN 03 | 74 |  | W506 | K508 | D542 |
|  | 13 | 0.23 | -1.35 | BEN 22 | 52 |  | P406 | P408 | R560 |
|  | 14 | 0.22 | -1.34 | BEN 19 | 54 | ** | L65 | I79 | F81 |
|  | 15 | 0.22 | -1.30 | BEN 20 | 54 |  | H290 | T440 | R442 |
|  | 16 | 0.22 | -1.30 | BEN 50 | 44 |  | Y31 | L35 | T61 |
|  | 17 | 0.22 | -1.30 | BEN 10 | 62 |  | P406 | L412 | L417 |
|  | 18 | 0.21 | -1.28 | BEN 32 | 50 |  | R178 | A312 | R339 |
|  | 19 | 0.21 | -1.27 | BEN 49 | 44 |  | V6 | F24 | R129 |
|  | 20 | 0.21 | -1.27 | BEN 42 | 46 |  | S464 | L438 | T440 |
|  | 21 | 0.21 | -1.26 | BEN 14 | 56 |  | R490 | R497 | I525 |
|  | 22 | 0.21 | -1.24 | BEN 06 | 66 |  | L455 | Y457 | V558 |
|  | 23 | 0.21 | -1.24 | BEN 31 | 50 |  | N117 | T532 | D534 |
|  | 24 | 0.20 | -1.21 | BEN 29 | 51 |  | V57 | Q62 | K73 |
|  | 25 | 0.20 | -1.19 | BEN 17 | 54 |  | I592 | R595 | N596 |
|  | 26 | 0.20 | -1.18 | BEN 04 | 67 |  | L455 | Y457 | N459 |
|  | 27 | 0.19 | -1.17 | BEN 28 | 51 |  | E128 | P238 | P242 |
|  | 28 | 0.19 | -1.16 | BEN 09 | 62 |  | W167 | L176 | K202 |
|  | 29 | 0.19 | -1.12 | BEN 36 | 48 |  | A237 | A389 | R392 |
|  | 30 | 0.19 | -1.12 | BEN 13 | 58 |  | I480 | A487 | Y515 |
|  | 31 | 0.19 | -1.11 | BEN 45 | 45 |  | K467 | Q586 | F587 |
|  | 32 | 0.19 | -1.11 | BEN 43 | 46 |  | V49 | P53 | D56 |
|  | 33 | 0.18 | -1.11 | BEN 24 | 51 |  | I35 | V103 | T104 |
|  | 34 | 0.18 | -1.09 | BEN 02 | 75 |  | H290 | K320 | R442 |
|  | 35 | 0.18 | -1.08 | BEN 25 | 51 |  | T416 | A553 | H554 |
|  | 36 | 0.18 | -1.05 | BEN 37 | 48 |  | K73 | K76 | P78 |
|  | 37 | 0.17 | -1.03 | BEN 39 | 47 |  | R22 | F24 | P234 |
|  | 38 | 0.16 | -0.97 | BEN 30 | 51 |  | R248 | I249 | P254 |
|  | 39 | 0.16 | -0.97 | BEN 26 | 51 |  | H245 | Y277 | I432 |
|  | 40 | 0.16 | -0.95 | BEN 23 | 52 |  | R186 | Y217 | K218 |
|  | 41 | 0.16 | -0.94 | BEN 44 | 45 |  | K28 | D32 | Q88 |
|  | 42 | 0.16 | -0.94 | BEN 33 | 50 |  | I20 | R21 | T231 |
|  | 43 | 0.15 | -0.90 | BEN 21 | 52 |  | L158 | H164 | Y205 |
|  | 44 | 0.15 | -0.89 | BEN 47 | 44 |  | V484 | I488 | A520 |
|  | 45 | 0.15 | -0.88 | BEN 38 | 48 |  | L240 | R427 | L428 |
|  | 46 | 0.13 | -0.78 | BEN 40 | 47 |  | E418 | K430 | Y457 |
|  | 47 | 0.13 | -0.75 | BEN 27 | 51 |  | A454 | L455 | K460 |
|  | 48 | 0.12 | -0.75 | BEN 41 | 46 |  | R303 | T367 | K394 |
|  | 49 | 0.11 | -0.65 | BEN 18 | 54 | ** | L92 | Y93 | D95 |
|  | 50 | 0.07 | -0.41 | BEN 35 | 48 |  | K477 | T550 | R579 |
| REF_2xzl | 1 | 0.22 | -1.35 | REF_15 | N/A |  | H290 | T440 | R442 |
| REF_2xzl | 2 | 0.22 | -1.32 | REF_01 | N/A |  | Y180 | P408 | T410 |
| REF_2xzl | 3 | 0.21 | -1.28 | REF_09 | N/A |  | R178 | S310 | R339 |
| REF_2xzl | 4 | 0.20 | -1.23 | REF_16 | N/A |  | T286 | R442 | R443 |
| REF_2xzl | 5 | 0.19 | -1.13 | REF_02 | N/A |  | R409 | L412 | V413 |
| REF_2xzl | 6 | 0.18 | -1.10 | REF_03 | N/A |  | N179 | Y180 | T410 |
| REF_2xzl | 7 | 0.18 | -1.09 | REF_06 | N/A |  | N177 | R178 | E201 |
| REF_2xzl | 8 | 0.17585 | -1.06 | REF_07 | N/A |  | R178 | N179 | P404 |
| REF_2xzl | 9 | 0.17 | -1.03 | REF_08 | N/A |  | R178 | S310 | R339 |
| REF_2xzl | 10 | 0.17 | -1.00 | REF_11 | N/A |  | V181 | E197 | H230 |
| REF_2xzl | 11 | 0.16 | -0.96 | REF_10 | N/A |  | H311 | R339 | N361 |
| REF_2xzl | 12 | 0.14 | -0.87 | REF_04 | N/A |  | P175 | N177 | N178 |
| REF_2xzl | 13 | 0.14 | -0.82 | REF_05 | N/A |  | P408 | D534 | R560 |
| REF_2xzl | 14 | 0.13 | -0.81 | REF_13 | N/A |  | E197 | H230 | A336 |
| REF_2xzl | 15 | 0.11 | -0.64 | REF_12 | N/A |  | E197 | A336 | R339 |
| REF_2xzl | 16 | 0.11 | -0.63 | REF_14 | N/A |  | E197 | A336 | R339 |
| REF_5fps | 1 | 0.23 | -1.36 | REF_09 | N/A |  | S289 | A316 | K320 |
| REF_5fpy | 2 | 0.21 | -1.26 | REF_11 | N/A |  | Y180 | P408 | T410 |
| REF_2zjo | 3 | 0.21 | -1.24 | REF_04 | N/A |  | S289 | A316 | E375 |
| REF_2zjo | 4 | 0.21 | -1.23 | REF_02 | N/A |  | A312 | E375 | Q537 |
| REF_2zjo | 5 | 0.20 | -1.18 | REF_03 | N/A |  | A312 | A316 | E319 |
| REF_2zjo | 6 | 0.19 | -1.14 | REF_01 | N/A |  | A312 | E375 | Q537 |
| REF_4b71 | 7 | 0.17 | -1.05 | REF_07 | N/A |  | I121 | L122 | T125 |
| REF_4b71 | 8 | 0.14 | -0.83 | REF_08 | N/A |  | A117 | N116 | K414 |
| REF_5fpt | 9 | 0.13 | -0.79 | REF_10 | N/A |  | P335 | A336 | A362 |
| REF_3rvb | 10 | 0.09 | -0.56 | REF_06 | N/A |  | P335 | A336 | A362 |
| REF_3rvb | 11 | 0.09 | -0.56 | REF_05 | N/A |  | K139 | E383 | N361 |
| Nsp15 (endo RNase) | Site (N) | LE | dG (Kcal/mol) | BEN | Conts (N) | N/A | Res 1 | Res 2 | Res 3 |
|  | 1 | 0.35 | -2.07 | BEN_05 | 72 |  | V315 | K335 | D336 |
|  | 2 | 0.31 | -1.87 | BEN_02 | 76 |  | L50 | P94 | I97 |
|  | 3 | 0.31 | -1.86 | BEN_14 | 55 |  | L252 | K277 | V295 |
|  | 4 | 0.30 | -1.78 | BEN_01 | 76 |  | F204 | L215 | K260 |
|  | 5 | 0.28 | -1.68 | BEN_04 | 72 |  | K159 | I169 | K174 |
|  | 6 | 0.27 | -1.63 | BEN_03 | 74 |  | Q45 | K46 | W59 |
|  | 7 | 0.26 | -1.53 | BEN_23 | 51 |  | V183 | T121 | N140 |
|  | 8 | 0.25 | -1.51 | BEN_08 | 62 |  | Y194 | Q192 | V321 |
|  | 9 | 0.25 | -1.49 | BEN_13 | 55 |  | H15 | I64 | K65 |
|  | 10 | 0.24 | -1.45 | BEN_22 | 51 |  | I80 | I108 | L120 |
|  | 11 | 0.24 | -1.44 | BEN_09 | 58 |  | E192 | Y194 | T322 |
|  | 12 | 0.23 | -1.36 | BEN_07 | 66 |  | L215 | K257 | K260 |
|  | 13 | 0.23 | -1.36 | BEN_24 | 50 |  | D311 | L312 | S313 |
|  | 14 | 0.23 | -1.36 | BEN_06 | 68 |  | Y238 | F241 | S242 |
|  | 15 | 0.22 | -1.33 | BEN_15 | 54 |  | I64 | K65 | L163 |
|  | 16 | 0.22 | -1.33 | BEN_16 | 53 |  | D311 | L312 | S313 |
|  | 17 | 0.22 | -1.29 | BEN_11 | 56 |  | K159 | R207 | L299 |
|  | 18 | 0.22 | -1.29 | BEN_12 | 56 |  | E4 | E22 | P24 |
|  | 19 | 0.21 | -1.27 | BEN_25 | 50 |  | T49 | R91 | A93 |
|  | 20 | 0.20 | -1.21 | BEN_21 | 51 |  | M1 | F16 | Q20 |
|  | 21 | 0.19 | -1.15 | BEN_17 | 53 |  | I223 | Q229 | E234 |
|  | 22 | 0.19 | -1.15 | BEN_18 | 53 |  | F269 | F280 | V292 |
|  | 23 | 0.17 | -1.04 | BEN_19 | 52 |  | I269 | K205 | R207 |
|  | 24 | 0.17 | -1.01 | BEN_20 | 52 |  | A109 | E114 | I116 |
|  | 25 | 0.11 | -0.65 | BEN_10 | 57 |  | H250 | K290 | Y343 |
| Nsp16 (2'-O  MT) | Site (N) | LE | dG (Kcal/mol) | BEN | Conts (N) | PPI (**) | Res 1 | Res 2 | Res 3 |
|  | 1 | 0.42 | -2.51 | BEN_01 | 100 |  | K6933 | K6944 | F6947 |
|  | 2 | 0.40 | -2.40 | BEN_02 | 85 |  | L6898 | M6929 | F6947 |
|  | 3 | 0.32 | -1.94 | BEN_03 | 84 | ** | R6884 | L6887 | T6889 |
|  | 4 | 0.32 | -1.93 | BEN_10 | 63 |  | L6961 | K7012 | I7065 |
|  | 5 | 0.30 | -1.79 | BEN_05 | 69 |  | Q6850 | K7047 | F7048 |
|  | 6 | 0.28 | -1.71 | BEN_20 | 56 | ** | V6876 | P6878 | V7902 |
|  | 7 | 0.28 | -1.67 | BEN_11 | 60 |  | L6820 | K6822 | H6972 |
|  | 8 | 0.27 | -1.62 | BEN_25 | 49 |  | R6817 | I7017 | M7022 |
|  | 9 | 0.26 | -1.53 | BEN_09 | 64 |  | L6820 | K6822 | H6972 |
|  | 10 | 0.25 | -1.51 | BEN_12 | 59 |  | L6820 | K6822 | H6972 |
|  | 11 | 0.25 | -1.51 | BEN_23 | 52 |  | H6917 | Y6950 | K6958 |
|  | 12 | 0.25 | -1.47 | BEN_06 | 69 | ** | L6892 | V7086 | I7088 |
|  | 13 | 0.24 | -1.47 | BEN_17 | 57 |  | L6855 | T6856 | W6987 |
|  | 14 | 0.24 | -1.45 | BEN_16 | 57 |  | P6888 | P7049 | L7052 |
|  | 15 | 0.24 | -1.43 | BEN_08 | 64 |  | P6860 | Y6861 | V7057 |
|  | 16 | 0.24 | -1.42 | BEN_21 | 55 |  | H6917 | A6919 | V7087 |
|  | 17 | 0.23 | -1.38 | BEN_14 | 58 |  | A6919 | R7085 | V7087 |
|  | 18 | 0.23 | -1.36 | BEN_24 | 51 | ** | A6881 | R6884 | D6904 |
|  | 19 | 0.22 | -1.34 | BEN_15 | 58 |  | K6921 | L6961 | Y7009 |
|  | 20 | 0.22 | -1.30 | BEN_18 | 57 |  | N6862 | L6892 | V7086 |
|  | 21 | 0.20 | -1.19 | BEN_13 | 58 |  | W6803 | Y7040 | F7043 |
|  | 22 | 0.19 | -1.16 | BEN_07 | 66 |  | W6803 | Y7040 | F7043 |
|  | 23 | 0.18 | -1.09 | BEN_19 | 57 |  | A6976 | K6980 | Y7020 |
|  | 24 | 0.16 | -0.95 | BEN_04 | 79 |  | L6825 | Y6828 | S7000 |
|  | 25 | 0.15 | -0.88 | BEN_22 | 53 |  | D6928 | M6929 | Y6930 |
| S2 Spike | Site (N) | LE | dG (Kcal/mol) | BEN | Conts (N) | N/A | Res 1 | Res 2 | Res 3 |
|  | 1 | 0.34 | -2.06 | BEN_01 | 83 |  | H1101.SEG2 | I1114.SEG2 | Y1138.SEG2 |
|  | 2 | 0.33 | -1.97 | BEN_05 | 69 |  | W886.SEG3 | V1040.SEG2 | Y1047.SEG2 |
|  | 3 | 0.33 | -1.95 | BEN_04 | 75 |  | K790.SEG3 | L806.SEG3 | P807.SEG3 |
|  | 4 | 0.32 | -1.91 | BEN_06 | 68 |  | W886.SEG2 | V1040.SEG1 | Y1047.SEG1 |
|  | 5 | 0.31 | -1.88 | BEN_12 | 64 |  | L1024.SEG3 | F1042.SEG3 | A1026.SEG1 |
|  | 6 | 0.31 | -1.84 | BEN_02 | 77 |  | P792.SEG1 | F797.SEG1 | P897.SEG1 |
|  | 7 | 0.31 | -1.84 | BEN_09 | 66 |  | P897.SEG3 | P1079.SEG2 | I1130.SEG2 |
|  | 8 | 0.31 | -1.83 | BEN_14 | 63 |  | W886.SEG1 | V1040.SEG3 | Y1047.SEG3 |
|  | 9 | 0.30 | -1.80 | BEN_25 | 58 |  | P897.SEG1 | P1079.SEG3 | I1130.SEG3 |
|  | 10 | 0.30 | -1.78 | BEN_36 | 55 |  | I726.SEG2 | I934.SEG2 | L938.SEG2 |
|  | 11 | 0.29 | -1.75 | BEN_07 | 67 |  | P897.SEG1 | P1079.SEG3 | I1130.SEG3 |
|  | 12 | 0.29 | -1.74 | BEN_19 | 60 |  | A930.SEG3 | K933.SEG3 | I932.SEG3 |
|  | 13 | 0.29 | -1.74 | BEN_20 | 59 |  | V796.SEG2 | L777.SEG2 | L966.SEG2 |
|  | 14 | 0.29 | -1.73 | BEN_50 | 51 |  | Q804.SEG1 | N928.SEG1 | I931.SEG1 |
|  | 15 | 0.29 | -1.72 | BEN_13 | 64 |  | V796.SEG2 | L777.SEG2 | L966.SEG2 |
|  | 16 | 0.28 | -1.70 | BEN_46 | 53 |  | I726.SEG3 | I934.SEG3 | L938.SEG3 |
|  | 17 | 0.28 | -1.70 | BEN_29 | 57 |  | A930.SEG2 | K933.SEG2 | I932.SEG2 |
|  | 18 | 0.28 | -1.69 | BEN_45 | 53 |  | L966.SEG1 | S967.SEG1 | V976.SEG1 |
|  | 19 | 0.28 | -1.66 | BEN_21 | 58 |  | S758.SEG3 | K964.SEG2 | S967.SEG2 |
|  | 20 | 0.28 | -1.66 | BEN_15 | 63 |  | A942.SEG2 | L945.SEG2 | Q949.SEG2 |
|  | 21 | 0.28 | -1.66 | BEN_10 | 65 |  | P792.SEG3 | F797.SEG3 | P897.SEG3 |
|  | 22 | 0.27 | -1.64 | BEN_24 | 58 |  | H1083.SEG2 | Y1138.SEG2 | Q1142.SEG2 |
|  | 23 | 0.27 | -1.61 | BEN_22 | 58 |  | H1083.SEG3 | Y1138.SEG3 | Q1142.SEG3 |
|  | 24 | 0.27 | -1.59 | BEN_11 | 64 |  | P792.SEG2 | F797.SEG2 | P897.SEG2 |
|  | 25 | 0.26 | -1.58 | BEN_27 | 57 |  | K795.SEG3 | L806.SEG3 | P807.SEG3 |
|  | 26 | 0.26 | -1.57 | BEN_31 | 56 |  | A893.SEG3 | Y789.SEG3 | K790.SEG3 |
|  | 27 | 0.26 | -1.56 | BEN_03 | 76 |  | P792.SEG2 | F797.SEG2 | P897.SEG2 |
|  | 28 | 0.26 | -1.56 | BEN_37 | 55 |  | E1092.SEG1 | Q1106.SEG1 | R1107.SEG1 |
|  | 29 | 0.26 | -1.56 | BEN_49 | 51 |  | Y756.SEG2 | F970.SEG1 | R995.SEG1 |
|  | 30 | 0.26 | -1.55 | BEN_18 | 60 |  | H1083.SEG1 | Y1138.SEG1 | Q1142.SEG2 |
|  | 31 | 0.26 | -1.55 | BEN_28 | 57 |  | K811.SEG1 | P812.SEG1 | R815.SEG1 |
|  | 32 | 0.26 | -1.53 | BEN_35 | 55 |  | K921.SEG1 | A924.SEG1 | I1130.SEG3 |
|  | 33 | 0.26 | -1.53 | BEN_30 | 57 |  | K921.SEG2 | A924.SEG2 | I1130.SEG1 |
|  | 34 | 0.25 | -1.48 | BEN_17 | 61 |  | K921.SEG1 | V1128.SEG3 | I1130.SEG3 |
|  | 35 | 0.25 | -1.47 | BEN_08 | 66 |  | H1083.SEG3 | Y1138.SEG3 | Q1142.SEG3 |
|  | 36 | 0.24 | -1.46 | BEN_47 | 52 |  | K790.SEG3 | L806.SEG3 | P807.SEG3 |
|  | 37 | 0.24 | -1.43 | BEN_39 | 55 |  | T739.SEG3 | M740.SEG3 | D745.SEG3 |
|  | 38 | 0.24 | -1.41 | BEN_16 | 62 |  | L1145.SEG1 | L1145.SEG2 | L1145.SEG3 |
|  | 39 | 0.23 | -1.37 | BEN_23 | 58 |  | K921.SEG2 | V1128.SEG1 | I1130.SEG1 |
|  | 40 | 0.23 | -1.36 | BEN_32 | 56 |  | E748.SEG3 | L981.SEG3 | S982.SEG3 |
|  | 41 | 0.22 | -1.35 | BEN_40 | 55 |  | E748.SEG1 | L981.SEG1 | S982.SEG1 |
|  | 42 | 0.22 | -1.31 | BEN_42 | 54 |  | K811.SEG3 | P812.SEG3 | R815.SEG3 |
|  | 43 | 0.22 | -1.30 | BEN_26 | 57 |  | P728.SEG3 | K947.SEG3 | V951.SEG3 |
|  | 44 | 0.21 | -1.26 | BEN_38 | 55 |  | S721.SEG1 | A930.SEG1 | K933.SEG1 |
|  | 45 | 0.21 | -1.25 | BEN_33 | 56 |  | I726.SEG3 | A944.SEG3 | K947.SEG3 |
|  | 46 | 0.21 | -1.25 | BEN_34 | 56 |  | A893.SEG2 | Y789.SEG2 | K790.SEG2 |
|  | 47 | 0.20 | -1.23 | BEN_41 | 55 |  | K786.SEG3 | L1034.SEG3 | K1045.SEG2 |
|  | 48 | 0.17 | -1.04 | BEN_48 | 52 |  | F817.SEG3 | D820.SEG3 | L821.SEG3 |
|  | 49 | 0.16 | -0.95 | BEN_43 | 54 |  | F817.SEG2 | D820.SEG2 | L821.SEG2 |
|  | 50 | 0.16 | -0.95 | BEN_44 | 53 |  | K795.SEG1 | P807.SEG1 | P809.SEG1 |
| REF_5t6s | 1 | 0.27 | -1.62 | REF 09 | N/A |  | S721.SEG2 | A930.SEG2 | K933.SEG2 |
| REF_5t6s | 2 | 0.27 | -1.60 | REF 10 | N/A |  | T724.SEG2 | I934.SEG2 | L938.SEG2 |
| REF_5t6s | 3 | 0.26 | -1.57 | REF 07 | N/A |  | S721.SEG1 | A930.SEG1 | K933.SEG1 |
| REF_5t6s | 4 | 0.26 | -1.55 | REF 11 | N/A |  | S721.SEG3 | A930.SEG3 | K933.SEG3 |
| REF_5t6s | 5 | 0.24 | -1.42 | REF 12 | N/A |  | T724.SEG3 | I934.SEG3 | L938.SEG3 |
| REF_5t6s | 6 | 0.23 | -1.39 | REF 08 | N/A |  | T724.SEG1 | I934.SEG1 | L938.SEG1 |
| REF_5t6s | 7 | 0.20 | -1.19 | REF 02 | N/A |  | A1020.SEG1 | L1024.SEG1 | N1023.SEG2 |
| REF_5t6s | 8 | 0.09 | -0.56 | REF 04 | N/A |  | L1024.SEG2 | F1042.SEG2 | T1027.SEG3 |
| REF_5t6s | 9 | N/A | 2.46 | REF 06 | N/A |  | L727.SEG3 | L1024.SEG3 | K1028.SEG3 |
| REF_5t6s | 10 | N/A | 2.54 | REF 03 | N/A |  | T1027.SEG2 | E1031.SEG2 | F1042.SEG2 |
| REF_5t6s | 11 | N/A | 2.56 | REF 01 | N/A |  | T1027.SEG1 | E1031.SEG1 | F1042.SEG1 |
| REF_5t6s | 12 | N/A | 3.35 | REF 05 | N/A |  | T1027.SEG3 | E1031.SEG3 | F1042.SEG3 |
